# 4-Hydroxy-nonenal—A Bioactive Lipid Peroxidation Product †

**DOI:** 10.3390/biom5042247

**Published:** 2015-09-30

**Authors:** Rudolf J. Schaur, Werner Siems, Nikolaus Bresgen, Peter M. Eckl

**Affiliations:** 1 Institute of Molecular Biosciences, University of Graz, Heinrichstrasse 33a, 8010 Graz, Austria; 2 Institute for Medical Education, KortexMed GmbH, Hindenburgring 12a, 38667 Bad Harzburg, Germany; E-Mail: siems@kortexmed.de; 3 Division of Genetics, Department of Cell Biology, University of Salzburg, Hellbrunnerstasse 34, 5020 Salzburg, Austria; E-Mail: nikolaus.bresgen@sbg.ac.at; 4 Division of Genetics, Department of Cell Biology, University of Salzburg, Hellbrunnerstasse 34, 5020 Salzburg, Austria; E-Mail: peter.eckl@sbg.ac.at

**Keywords:** 4-hydroxy-nonenal, HNE, n-6 polyunsaturated fatty acids, Michael adducts, mercapturic acid

## Abstract

This review on recent research advances of the lipid peroxidation product 4-hydroxy-nonenal (HNE) has four major topics: I. the formation of HNE in various organs and tissues, II. the diverse biochemical reactions with Michael adduct formation as the most prominent one, III. the endogenous targets of HNE, primarily peptides and proteins (here the mechanisms of covalent adduct formation are described and the (patho-) physiological consequences discussed), and IV. the metabolism of HNE leading to a great number of degradation products, some of which are excreted in urine and may serve as non-invasive biomarkers of oxidative stress.

**Table of Contents**
**Preface…………………………………………………………………………………………………………………………………****2251****1. Lipid Peroxidation as a Free Radical Amplification Process…………………………………………………………………****2252****2. Structure, Properties and Generation of HNE…………………………………………………………………………………****2255****3. Major Reaction Mechanisms……………………………………………………………………………………………………****2257** 3.1. Reactions of the C=C Double Bond……………………………………………………………………………………………2257  3.1.1. Michael Additions…………………………………………………………………………………………………………2257  3.1.2. Reduction…………………………………………………………………………………………………………………2258  3.1.3. Epoxidation………………………………………………………………………………………………………………2259 3.2. Reactions of the Carbonyl Group……………………………………………………………………………………………2259  3.2.1. Acetal and Thio-Acetal Formation………………………………………………………………………………………2259  3.2.2. Schiff-Base Formation……………………………………………………………………………………………………2259  3.2.3. Oxidation…………………………………………………………………………………………………………………2259  3.2.4. Reduction…………………………………………………………………………………………………………………2260 3.3. Reactions of the Hydroxy Group………………………………………………………………………………………………2262**4. Biophysical Effects………………………………………………………………………………………………………………****2262****5. Biochemical Targets of HNE……………………………………………………………………………………………………****2262** 5.1. Reactions with Peptides and Proteins…………………………………………………………………………………………2263  5.1.1. Substrates…………………………………………………………………………………………………………………2265  5.1.1.1. Glutathione………………………………………………………………………………………………………………2265  5.1.1.2. Carnosine………………………………………………………………………………………………………………2267  5.1.1.3. Thioredoxin……………………………………………………………………………………………………………2267  5.1.1.4. Cytochrome c……………………………………………………………………………………………………………2268  5.1.2. Enzymes…………………………………………………………………………………………………………………2268  5.1.2.1. Oxidoreductases…………………………………………………………………………………………………………2269  5.1.2.1.1. Lactate Dehydrogenase………………………………………………………………………………………………2269  5.1.2.1.2. Glyceraldehyde-3-Phosphate Dehydrogenase (GAPDH)…………………………………………………………2269  5.1.2.2. Transferases……………………………………………………………………………………………………………2270  5.1.2.2.1. Glutathione-S-Transferase (GST)……………………………………………………………………………………2270  5.1.2.2.2. Liver Kinase B1 (LKB1)………………………………………………………………………………………………2270  5.1.2.2.3. 5'-AMP-Activated Protein Kinase (AMPK)…………………………………………………………………………2271  5.1.2.2.4. ZAK Kinase (Sterile Alpha Motif and Leucine Zipper Containing Kinase AZK)…………………………………2271  5.1.2.2.5. Serine/Threonine-Protein Kinase AKT2 (Proteinkinase B2)………………………………………………………2271  5.1.2.3. Hydrolases………………………………………………………………………………………………………………2271  5.1.2.3.1. ATP Synthase…………………………………………………………………………………………………………2271  5.1.2.3.2. Phosphatase and Tensin Homolog Deleted on Chromosome 10 (PTEN)……………………………………………2272  5.1.2.3.3. Sirtuin 3 (SIRT3)……………………………………………………………………………………………………2272  5.1.2.3.4. Cathepsins……………………………………………………………………………………………………………2273  5.1.2.3.5. Neprilysin (NEP)……………………………………………………………………………………………………2273  5.1.2.4. Lyases……………………………………………………………………………………………………………………2273  5.1.2.4.1. Mitochondrial Aconitase (ACO2)……………………………………………………………………………………2273  5.1.2.4.2. α-Enolase………………………………………………………………………………………………………………2273  5.1.2.5. Isomerases………………………………………………………………………………………………………………2274  5.1.2.5.1. Protein Disulfide Isomerase (PDI)……………………………………………………………………………………2274  5.1.2.5.2. Peptidyl-Prolyl *Cis*/*Trans*-Isomerase A1 (Pin1)………………………………………………………………………2274  5.1.2.6. Ligases: Glutamine Synthetase…………………………………………………………………………………………2274  5.1.3. Carriers……………………………………………………………………………………………………………………2274  5.1.3.1. Albumin…………………………………………………………………………………………………………………2274  5.1.3.2. Hemoglobin and Myoglobin……………………………………………………………………………………………2275  5.1.3.3. Liver and Adipocyte Fatty Acid-Binding Protein (FABP)……………………………………………………………2275  5.1.3.4. Apolipoprotein B-100 (ApoB)…………………………………………………………………………………………2275  5.1.3.5. β-Lactoglobulin…………………………………………………………………………………………………………2276  5.1.4. Transporters and Channels………………………………………………………………………………………………2276  5.1.4.1. Glutamate Transport Protein……………………………………………………………………………………………2276  5.1.4.2. α-Synuclein (α-Syn)……………………………………………………………………………………………………2276  5.1.4.3. Sarco/Endoplasmic Reticulum Ca^2+^-ATPase (SERCA1a)……………………………………………………………2277  5.1.4.4. Transient Receptor Potential Vanilloid 1 (TRPV1)……………………………………………………………………2277  5.1.4.5. Dopamine Transporter…………………………………………………………………………………………………2278  5.1.5. Receptors…………………………………………………………………………………………………………………2278  5.1.5.1. Platelet-Derived Growth Factor Receptor-β (PDGFR-β)………………………………………………………………2278  5.1.5.2. Lectin-Like Oxidized Low-Density Lipoprotein Receptor-1 (LOX-1)………………………………………………2278  5.1.5.3. Toll-Like Receptor 4 (TLR4)……………………………………………………………………………………………2278  5.1.6. Cytoskeletal Proteins………………………………………………………………………………………………………2279  5.1.6.1. Tau Proteins……………………………………………………………………………………………………………2279  5.1.6.2. Ankyrin…………………………………………………………………………………………………………………2279  5.1.6.3. Spectrins…………………………………………………………………………………………………………………2280  5.1.7. Chaperones: Heat Shock Proteins 70 and 90……………………………………………………………………………2280  5.1.8. Uncoupling Proteins 2 and 3 (UCP2 and UCP3)…………………………………………………………………………2282  5.1.9. Growth Factors: Platelet-Derived Growth Factor (PDGF)………………………………………………………………2283  5.1.10. Peptide Hormones………………………………………………………………………………………………………2283  5.1.10.1. Insulin…………………………………………………………………………………………………………………2283  5.1.10.2. Angiotensin II…………………………………………………………………………………………………………2283  5.1.11. Extracellular Matrix Proteins: Collagen…………………………………………………………………………………2283  5.1.12. Histones: Histone-H2A…………………………………………………………………………………………………2284 5.2. Reactions with Lipids…………………………………………………………………………………………………………2284 5.3. Reactions with Cofactors and Vitamins………………………………………………………………………………………2284  5.3.1. Vitamin C (Ascorbic Acid)………………………………………………………………………………………………2284  5.3.2. Pyridoxamine……………………………………………………………………………………………………………2285  5.3.3. Lipoic Acid………………………………………………………………………………………………………………2285 5.4. Reactions with Nucleic Acids…………………………………………………………………………………………………2285**6. Formation of HNE in Mammalian Cells and Tissues…………………………………………………………………………****2287** 6.1. HNE Formation in Cellular and Organ Systems………………………………………………………………………………2287 6.2. HNE in the Whole Healthy Organism…………………………………………………………………………………………2289 6.3. Influence of Nutrition…………………………………………………………………………………………………………2290**7. Metabolism of HNE………………………………………………………………………………………………………………****2291** 7.1. HNE Metabolism in Mammalian Cells and Organs…………………………………………………………………………2293 7.2. HNE Metabolism in Subcellular Organelles………………………………………………………………………………… 2294 7.3. HNE Metabolism in Whole Animals and Interorgan Relationships…………………………………………………………2295 7.4. Primary HNE Intermediates—Enzymatic Reactions and Quantitative Results………………………………………………2295 7.5. Secondary HNE Intermediates—Enzymatic Reactions and Quantitative Results……………………………………………2301 7.6. HNE Metabolism as a Component of the Antioxidative Defense System……………………………………………………2306 7.7. HNE Intermediates as Potential Biomarkers of LPO…………………………………………………………………………2307 7.8. Further Medical Applications of HNE Metabolism…………………………………………………………………………2307**8. Conclusions………………………………………………………………………………………………………………………****2309****Conflicts of Interest…………………………………………………………………………………………………………………****2309****Abbreviations………………………………………………………………………………………………………………………****2309****References……………………………………………………………………………………………………………………………****2313****Tables and Figures**
Table 1. HNE concentrations in cells, tissues and organs……………………………………………………………………………2289Table 2. Maximal velocity of total HNE degradation in cells, subcellular organelles, and perfused organs…………………………………………………………………………………………………………………………………2292Table 3. Primary HNE metabolites in different cells and tissues after the addition of 100 μM HNE to the biological system…………………………………………………………………………………………………………………………………2301Figure 1. Idealized representation of the initiation and propagation reactions of lipid peroxidation2253Figure 2. Formation of lipid hydroperoxides and cyclic peroxides from arachidonic acid.…………………………………………2254Figure 3. Chemical structure of 4-hydroxy-2-trans-nonenal (HNE)…………………………………………………………………2255Figure 4. Overview of the reactions of 4-hydroxy-nonenal with different biomolecules……………………………………………2257Figure 5. HNE plasma concentration in dependence on age of the blood donor (5 to 90 years)……………………………………2288Figure 6. Degradation/metabolism of 4-HNE in rat hepatocytes……………………………………………………………………2292Figure 7. Identification of HNE and 4-hydroxynonenoic acid (HNA) by isocratic HPLC separation………………………………2297Figure 8. Mass spectrum of dihydroxynonene urethane (HPLC plus MS) with fluorimetric detection……………………………2298Figure 9. HNE metabolites.……………………………………………………………………………………………………………2299Figure 10. HPLC chromatogram of the isoindol derivative of the HNE-GSH conjugate (reaction product in presence of o-phthalaldehyde)………………………………………………………………………………………………………………………2306

## Preface

Due to its diverse biochemical reactions and far-reaching biological effects 4-hydroxy-nonenal (HNE) is a fascinating compound, the discovery of which is hidden deep in the scientific literature. When Hermann Esterbauer isolated this aldehyde for the first time a quantitative analysis of its chemical elements was made resulting in values in between those for 4-hydroxy-octenal and 4-hydroxy-nonenal (Percent Composition: C 69.19%, H 10.32%, O 20.48%). Since these values were closer to those of 4-hydroxy-octenal Esterbauer and his supervisor Schauenstein published it with this chemical formula [1]. Only later the experimental error became evident. Thus, if one searches the literature for 4-hydroxy-nonenal, one would hardly find the paper describing its discovery as a product of lipid peroxidation.

Lipid peroxidation is a prominent feature of oxidative stress (for a review see the classical monograph of Halliwell and Gutteridge [2]), to which reference is made here. Additionally, a multi-author work on lipid peroxidation was edited by Catala [3]. Lipid peroxidation is a free radical amplification process yielding a great variety of bioactive products. One of the most extensively studied examples is HNE, which will be treated in this review. Since several reviews on HNE appeared previously [4,5,6], the focus of this contribution is directed on recent advances. In a recent review Fritz and Petersen [7] describe the generation and chemical reactivity of HNE and other lipid-derived aldehydes with a special attention to the homeostatic responses to electrophilic stress. Zarkovic *et al.* [8] describe the pathophysiological relevance of aldehydic protein modifications with special attention to HNE. A recent review of Perluigi *et al.* [9] describes the role of lipid peroxidation, particularly of HNE-induced protein modification, in neurodegenerative diseases. Furthermore, a review of Riahi *et al.* [10] addresses the origin and cellular functions of 4-hydroxyalkenals. Forman [11] reviewed the role of HNE and other α,β-unsaturated aldehydes as well as reactive oxygen species in signal transduction. New concepts and molecular mechanisms in cell signalling by reactive lipid species are discussed by Higdon *et al.* [12]. Similarly, Dwivedi *et al.* [13] as well as Ayala *et al.* [14] describe HNE as a signaling molecule. The chemistry and analysis of HNE was reviewed by Spickett [15]. LoPachin *et al.* [16] discussed the chemical attributes of HNE and acrolein that determine their toxicities.

A review of Catala [3] provide a synopsis of identified effects of HNE and other hydroxy-alkenals and oxidized phospholipids on cell signaling, from their intracellular production to their action as intracellular messengers, up to their influence on transcription factors and gene expression. Mattson [17] gave an overview of the roles of HNE in obesity, the metabolic syndrome, and associated vascular and neurodegenerative disorders. Finally, Guichardant and Lagarde [18] reviewed HNE as well as 4-hydroxy-hexenal and their degradation products as biomarkers of oxidative stress.

## 1. Lipid Peroxidation as a Free Radical Amplification Process

In this chapter a brief overview will be presented on the molecular mechanisms underlying lipid peroxidation. Lipid peroxidation was defined by Tappel [19] as the *oxidative deterioration of polyunsaturated lipids*. Poly-unsaturated fatty acids are those that contain two or more carbon-carbon double bonds. Initiation of lipid peroxidation can be caused by addition of a reactive species (RS) or, *more usually*, by hydrogen atom abstraction from a methylene (-CH_2_-) group by a RS; in both cases, a carbon radical results. For example, the hydroxyl radical ^●^OH can react by addition (Equation (1)) or by H^●^ abstraction (Equation (2)).

-HC=CH- + ^●^OH → -HC(OH)-^●^CH- (1)

-CH_2_- + ^●^OH → -^●^CH- + H_2_O (2)

**Figure 1:**
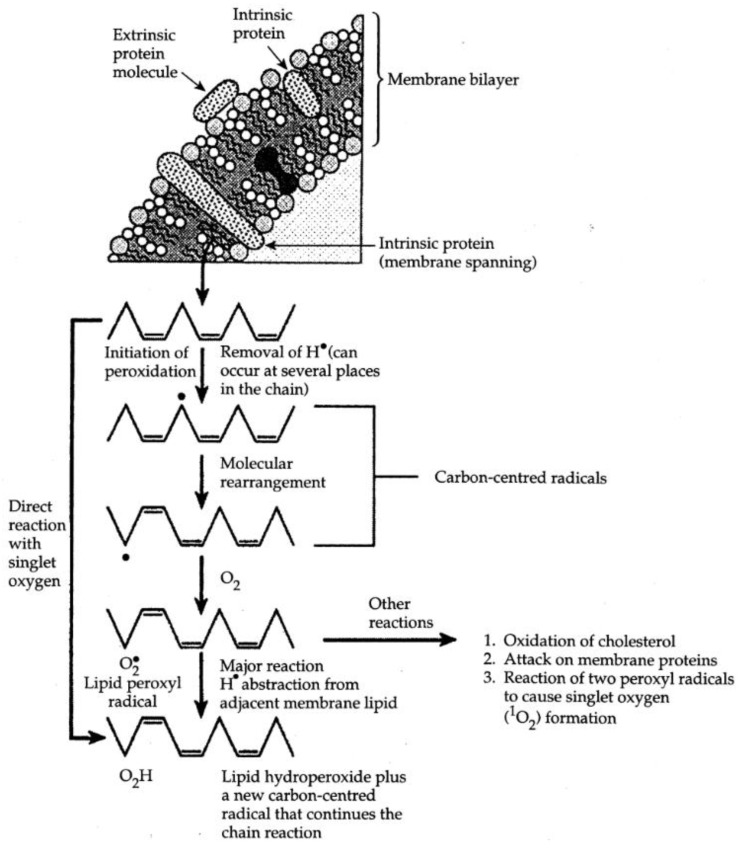
Idealized representation of the initiation and propagation reactions of lipid peroxidation. The peroxidation of fatty acids with three double bonds is shown. Reprinted from [2].

A double bond weakens the bond energy of the C-H bonds present on the next carbon atom (the allylic hydrogens), especially when there is a double bond on either side of the C-H bond (Figure 1), giving bis-allylic hydrogens.

Hydroxyl radicals readily initiate peroxidation of fatty acids, lipoproteins and membranes. In addition the hydroperoxyl radical HOO^●^ can abstract H from some polyunsaturated fatty acids (Equation (3))

HOO^●^ + CH → HOOH + C^●^ (3)

and it can stimulate peroxidation by reaction with preformed lipid hydroperoxides to generate peroxyl radicals [20] (Equation (4)).

HOO^●^ + ROOH → ROO^●^ + H_2_O_2_ (4)

The carbon radicals often stabilize by molecular rearrangement to form conjugated dienes (Figure 1).

Under aerobic conditions carbon radicals readily combine with O_2_ to give a peroxyl radical (Equation (5)).

R^●^ + O_2_ → ROO^●^ (5)

Peroxyl radicals can also abstract H^●^ from an adjacent fatty-acid side-chain (Equation (6)).

ROO^●^ + CH → ROOH + C^●^ (6)

This is the propagation stage of lipid peroxidation. It forms new carbon radicals that can react with O_2_ to form new peroxyl radicals, and so the chain reaction of lipid peroxidation continues (Figure 1). The ROO^●^ combines with the H^●^ that it abstracts to give a lipid hydroperoxide (ROOH). A single initiation event thus has the potential to generate multiple peroxide molecules by a chain reaction.

**Figure 2:**
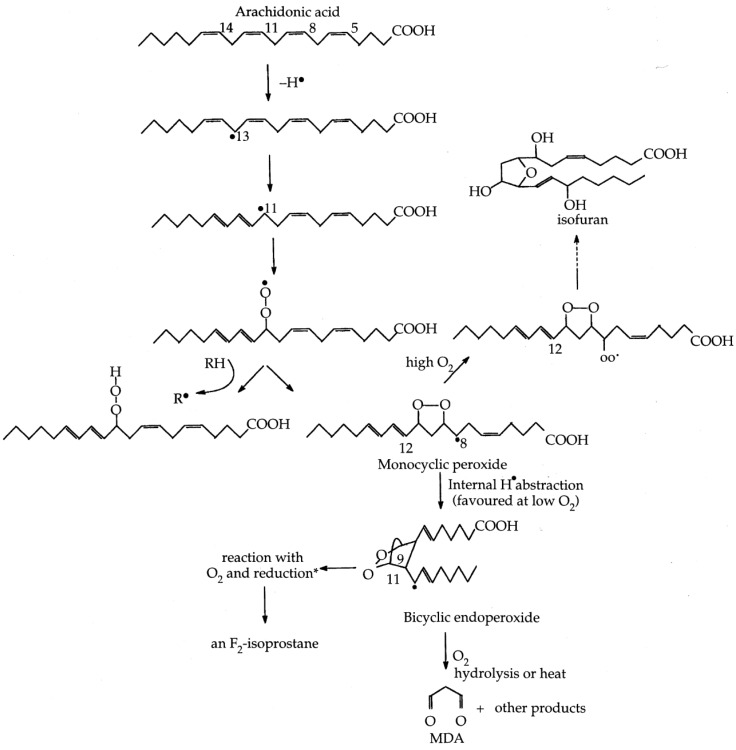
Formation of lipid hydroperoxides and cyclic peroxides from arachidonic acid. The chemistry is complex because abstraction of H^●^ can occur at different sites; for simplicity we show it only for C13. The ROO^●^ radicals formed can generate hydroperoxides or cyclize to monocyclic peroxides. This results when peroxyl radicals attack a double bond in the same chain. Monocyclic peroxides can form bicyclic structures that can give rise to isoprostanes, or react with O_2_, eventually producing isofurans. Unstable peroxides can decompose to aldehydes such as malondialdehyde. Even products containing bicyclic endoperoxide and cyclic peroxide groups in the same molecule (dioxolane-isoprostanes) can be generated [23]. O_2_ concentration affects the relative levels of isoprostanes and other products. Reprinted from [2].

Cyclic and bicyclic peroxides can also form (Figure 2).

Peroxidized lipids are bioactive. They can have effects on cells similar to those of H_2_O_2_: low levels can stimulate proliferation, higher levels block proliferation and yet higher ones induce apoptosis and necrosis [21]. Such events occur in atherosclerosis and maybe in the gastrointestinal tract. Products of lipid peroxidation can also have more specific effects. For example, some mimic the actions of platelet activating factor (PAF = 1-*O*-alkyl-2-acetyl-sn-glycero-3-phospho-choline). Peroxidation of phosphatidyl-choline (lecithin) can generate fragments that bind to PAF receptors on target cells and exert PAF-like activity. Oxidized PAPC (1-palmitoyl-2-arachidonoyl-*sn*-glycero-3-phospho-choline) (ox-PAPC) can *stimulate* cytokine production in endothelial cells, but can *inhibit* activation of transcription factor NFκB by endotoxin and subsequent cytokine production, *i.e.*, it can be anti-inflammatory under some circumstances. It can also increase levels of haem-oxygenase, a cytoprotective enzyme in endothelial cells. Intraperitoneal injection of ox-PAPC allows mice that had been treated with a normally lethal dose of endotoxin, to survive [22]. Hence several specific lipid oxidation products act as signalling molecules and their actions are not always bad*.* It can be predicted that more such roles will be found.

## 2. Structure, Properties and Generation of HNE

**Figure 3:**
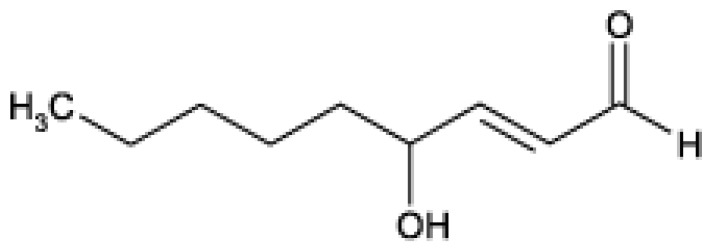
Chemical structure of 4-hydroxy-2-*trans*-nonenal (HNE).

The chemical structure of 4-hydroxy-2-*trans*-nonenal (4-hydroxy-2E-nonenal, HNE) is shown in Figure 3.

HNE is an extraordinarily reactive compound containing three functional groups which in many cases act in concert and help to explain its high reactivity [24]. Most importantly there is a conjugated system of a C=C double bond and a C=O carbonyl group which provide a partial positive charge to carbon 3, because this structure contains mobile π-electrons. The carbonyl oxygen atom is electronegative and can promote the withdrawal of mobile electron density from the beta-carbon atom causing regional electron deficiency. Considering this type of electron polarizability, HNE is considered to be a *soft* electrophile that preferentially forms 1,4-Michael type adducts with *soft* nucleophiles. Cysteine sulfhydryl groups are the primary soft nucleophilic targets of HNE while lysine and histidine residues are harder biological nucleophiles [16]. The positive charge on carbon 3 is further increased by the inductive effect of the hydroxy group at carbon 4. Therefore nucleophilic attack e.g., by thiol or amino groups occurs primarily at carbon 3 and secondarily at the carbonyl carbon 1.

The relationship between structure and activity has been studied in a toxicity test using L6 muscle cells [25]. The effect of 4-hydroxy-2-alkenals was compared to several chemically related derivatives in order to clarify the physico-chemical requirement of their toxicity in L6 muscle cells. The rank of derivative toxicity was: hydroxy-alkenals > acetal derivatives approximate to 2-alkenals > alkanals and a high correlation was found between toxicity and protein carbonylation. This suggests a cyto-protective effect of nucleophilic scavengers against electrophilic compounds which could be of potential therapeutic benefit in oxidative stress associated diseases.

For the biological effects of HNE it is essential that the lipophilic properties are more pronounced than its hydrophilic properties. Thus HNE tends to concentrate in biomembranes rather than in the aqueous space of cells. HNE can be easily transferred from a membrane to both cytosol and the extracellular space. Studying the behaviour within membranes by using the time-dependent fluorescence shift method and molecular dynamics simulations demonstrated the stabilization of HNE in the carbonyl region of a 1-palmitoyl-2-oleoyl-sn-glycero-3-phosphocholine bilayer [26]. Thus, HNE is able to react with cell membrane proteins and lipids albeit stabilization in the membrane is moderate and HNE shuttling to either the extra- or intracellular space occurs in the microsecond range. These characteristics of the HNE—lipid membrane interaction provide a good explanation for the observed reactivity of HNE with proteins inside and outside the cell.

HNE shows chirality at carbon 4. This might be also biologically relevant. Although HNE is formed in tissues as a racemate, enantiospecific HNE effects have not yet been widely investigated. Different cellular responses have been reported for treatment with (R)-HNE, (S)-HNE, or racemic HNE. For instance, (S)-HNE and racemic HNE potently stimulate phosphorylation of Jun kinase and Akt while (R)-HNE is strongest in phosphorylating MAPK. Also, (S)-HNE shows a more pronounced cytotoxicity [27,28] which points at the relevance of HNE enantiomers in cellular responses to HNE. Furthermore, Guéraud *et al.* [29] have shown, that (R)- and (S)-HNE are enantioselectively metabolised in rats and d_11_-4-hydroxy-2-(E)-nonenal is conjugated with glutathione in an enantio-selective mode, and exported from the liver [30].

Generally, HNE reacts with some sulfhydryl groups in proteins in a stereoselective manner. This has been characterized in detail by Wakita *et al.* [31]. Incubation of *N*-acetylcysteine with the S- and R-HNE entantiomer generated HNE-cystein adducts characterized as anomeric isomers by reverse-phase HPLC and NMR. The stereoselective formation of HNE-cyteine adducts was also demonstrated in the redox-regulatory protein thioredoxin, the active site at Cys^32^ showing a preference for R-HNE cysteine adducts. These findings provide insight into structural aspects of lipid peroxidation product/HNE—based sulfhydryl modification and the chemical characterization of protein S-associated aldehydes *in vitro* and *in vivo*. In this context it is worth to mention that an efficient, enantioselective synthesis of (R)- and (S)-HNE has been worked out recently by Komisarski *et al.* [32].

Of special relevance, HNE and its glutathione conjugates are able to regulate oxidative stress related transcription factors such as NFκB and AP-1 by addressing protein kinase cascade mediated stress signaling. This transcriptional activation leads to an upregulated expression of several genes involved in cell differentiation and cell death control. The mechanisms by which HNE and other lipid aldehydes transduce activation of NFκB signaling pathways have been described by Yadav *et al.* [33].

Regulatory effects of HNE can be based on purely metabolic mechanisms. Disruption of GST-based HNE conjugation, resulting in elevated HNE-levels, promotes obesity in mice and the nematode *Caenorhabditis elegans* suggesting a phylogenetically conserved, HNE-dependent mechanism involved in lipid metabolism [34,35]. In *C. elegans*, enhanced HNE-conjugation favours a lean phenotype while an inhibition of conjugation or oxidation of HNE yields fat accumulation. Together with the finding that synthetic HNE also stimulates lipid deposition in *C. elegans*, a causative role of HNE, acting on a metabolic basis in the development of an obese phenotype cannot be ruled out.

HNE is a degradation product of ω-6 polyunsaturated fatty acids, and the different mechanisms of HNE formation have been reviewed recently by Schneider *et al.* [36] and Spickett [15]. Notably, 4-hydroxy-hexenal (HHE), representing the corresponding hydroxy-alkenal derived from ω-3 polyunsaturated fatty acids shares several properties with HNE, but also shows important differences particularly with respect to targets of aldehyde adduct formation and detoxification pathways [37].

HNE formation may follow several radical-dependent oxidative routes involving the generation of hydroperoxides, alkoxyl radicals, epoxides, and fatty acyl cross-linking reactions. Cleavage of the oxidized fatty acyl chain results in HNE formation from the methyl end, and 9-oxo-nonanoic acid from the carboxylate or esterified end of the chain, although many other products are also possible.

Furthermore, the enzymatic production of HNE has been described by Wang *et al.* [38], reporting that *Escherichia faecalis*, a human intestinal commensal, triggers COX-2 based HNE formation in macrophages.

Finally, HNE is hepato- and nephrotoxic in mammalians [39]. Interestingly, high HNE concentrations (≥20 µM) are considered to contribute to cardiac ischemia-reperfusion injury, while lower, sublethal aldehyde concentrations (5 µM) serve cardioprotection by supporting cellular stress resistance [40]. At low concentrations, HNE stimulates the Nrf2 (NFE2-related factor 2) mediated upregulation of γ-glutamylcysteine ligase and the core subunit of the high-affinity cystine transporter Xc(-) yielding a 1.45-fold shift of intracellular GSH levels.

## 3. Major Reaction Mechanisms

**Figure 4:**
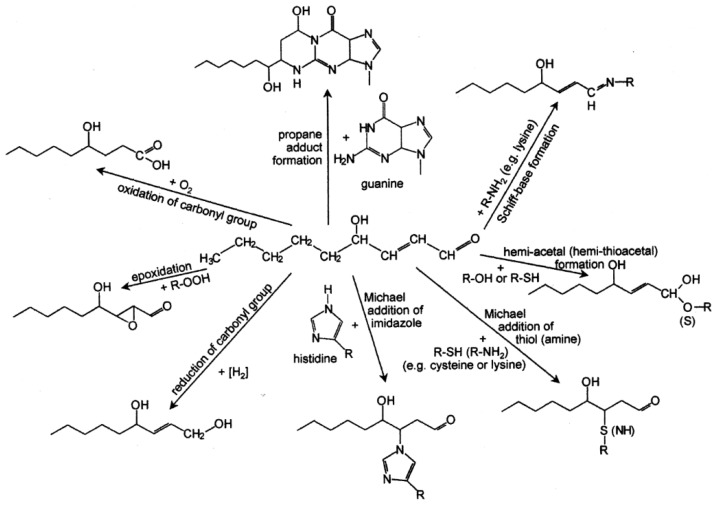
Overview of the reactions of 4-hydroxy-nonenal with different biomolecules. Explanations in the text. Reprinted from [24].

Essentially, HNE exhibits a high reactivity towards thiol and amino groups which are responsible for most of the biochemical effects of HNE. An overview of the reactions of HNE with different biomolecules is shown in Figure 4.

### 3.1. Reactions of the C=C Double Bond

#### 3.1.1. Michael Additions

In Michael additions a nucleophile, e.g., cysteine or glutathione (GSH), is added to the C=C double bond (Figure 4). In some cases the reaction is reversible (Retro Michael cleavage). In case of GSH the reaction velocity is greatly enhanced by the enzyme glutathione-S-transferase (GST) as reviewed by Mannervik (2012) [41]. In particular the GSTA4-4 isoform strongly catalyzes the reaction between HNE and GSH which is due to the high alkenal activity related to C-terminal interactions [42]. The elimination of an excess of HNE by GST activity may confer cytoprotection, however, this can change to the opposite in the presence of interacting factors such as the multidrug resistence protein-1 (MRP1). This is demonstrated by the HNE mediated cytotoxicity seen in MCF7 cells stably expressing MRP1 together with human GST-M1 (GSTM1) [43]. In these cells, the co-expression of GSTM1 and MRP1 leads to increased GSH depletion and formation of protein adducts upon HNE exposure which facilitated a cytotoxic outcome rather than protection.

GSTA4-4 was also used to address the question whether HNE metabolizing enzymes avoid HNE mediated inactivation [44]. Despite marked structural similarities between several GST isoforms (GSTA1-1, GSTA4-4, GSTP1-1) GST A4-4 shows the highest catalytic activity to metabolize HNE but is insensitive to covalent HNE adduct formation.

Similar to thiols amino compounds such as lysine, ethanol-amine, guanine and the imidazole group of histidine can also undergo Michael additions to HNE. For example, the histidine-containing dipeptide carnosine is a target of HNE [45].

As a recent example of Michael adduction of proteins by HNE, the modification of recombinant human phosphatase PTEN (Phosphatase and Tensin homolog) by HNE was studied by Shearn *et al.* [46] who identified several Michael adducts of HNE on Cys^71^, Cys^136^, Lys^147^, Lys^223^, Cys^250^, Lys^254^, Lys^313^, Lys^327^ and Lys^344^ by mass analysis (LC/MS/MS). HNE adducts to Cys^71^ adjacent to the active center and Lys^327^ locating to the C2 domain of PTEN may lead to PTEN inhibition via steric hindrance of the active-site pocket or abrogation of PTEN functions associated with the C2 domain. Shearn *et al.* [46] also propose that Akt2 could be activated by the HNE mediated inhibiton of PTEN which suggests a potential novel mechanism of lipid accumulation initiated by an increased production of reactive aldehydes during chronic ethanol administration.

In rare cases Michael addition reactions of HNE as well as acrolein (ACR) have been observed with aromatic hydroxy groups of polyphenols. The formation of adducts with HNE or ACR was reported for naturally occurring flavan-3-ols, theaflavins, cyanomaclurin, dihydrochalcones and phloretin which showed the most efficient scavenging activity [47]. With respect to this, certain polyphenols could be of benefit in antagonizing “carbonyl stress” via quenching of α,β-unsaturated aldehydes. Finally, Michael addition of HNE to ascorbic acid has also been described, targeting an activated C-H bond followed by ring closure and hemiacetal formation (*i.e.*, addition of a hydroxyl group to a carbonyl group) [31,48].

Several methods are available at present to study Michael adduction by HNE. In particular, polyclonal and monoclonal antibodies (e.g., applied as ELISA) are intensively used to assess Michael-adduct formation of HNE with proteins [49]. Michael addition-type amino acid adducts of HNE can also be readily detected by HPLC methodology employing 2-aminopyridine (2-AP) as fluorescent probe [50]. Moreover the relation between protein adduct formation and gene expression is studied by the integration of protein adduction, gene expression, protein-DNA interaction, and protein-protein interaction data following a systemic approach [51].

#### 3.1.2. Reduction

The C=C double bond can be reduced by an alkenal/one oxidoreductase with NAD(P)H as cofactor to yield 4-hydroxynonanal (Figure 4).This enzyme can metabolize HNE quite efficiently, which is demonstrated by the effect of NADP(H) overexpression seen in human embyonic kidney cells, efficiently protecting the cells from HNE challenge [52].

#### 3.1.3. Epoxidation

As further reaction, epoxidation in presence of a hydroperoxide may introduce an oxirane ring into HNE (Figure 4). Albeit the exact underlying mechanism for this reaction remains to be defined, epoxides are formed by the incubation of HNE with hydrogen peroxide or 9- or 13-linoleic acid hydrogen peroxide at 37 °C for 24 h as well as in the presence of a fatty acid (linoleic acid, γ-linolenic acid, or arachidonic acid) and lipoxygenase [53].

### 3.2. Reactions of the Carbonyl Group

#### 3.2.1. Acetal and Thio-Acetal Formation

The reaction of the carbonyl group of HNE with alcohols or thiols is a two step mechanism yielding (thio)acetals via (thio)hemi-actals (Figure 4). Since acetals are much more stable than HNE the reaction of HNE with methanol or ethanol is frequently used in synthetic chemistry for the storage of HNE. The free aldehyde can easily be released again in acidic medium. The hydroxyl group at carbon 4 of HNE can undergo a hemi-acetal formation only as a secondary reaction after a primary reaction such as a Michael addition which confers rotatory freedom to the C2-C3 bond.

#### 3.2.2. Schiff-Base Formation

Schiff-base formation with primary amines, e.g., lysine, is a competitive reaction to the Michael addition of amines (Figure 4). This reaction contributes frequently to the crosslinking of proteins by HNE as was observed in mouse sciatic nerves. It has been shown that HNE adducts characterize axons of the mouse sciatic nerve from birth to senility. Using an antibody specific for a lysine-lysine cross-link, Perry *et al.* [54] examined axonal labeling in these nerves. As demonstrated by immunoblotting, HNE mediated intramolecular cross-linking is restricted to heavy and medium neurofilament subunits, the extent of HNE modification remaining relatively constant over the life span. Furthermore, Schiff-base formation may also have therapeutic implications. This is suggested by the reaction of the HNE carbonyl group with the hydrazine functional group of *phenelzine* underlying the HNE scavenging effect of this agent in traumatic brain injury [55].

#### 3.2.3. Oxidation

The oxidation of the HNE carbonyl group producing 4-hydroxy-nonenoic acid is catalyzed by aldehyde dehydrogenase (ALDH), NAD^+^ or NADP^+^ acting as cofactor (Figure 4). Fournet *et al.*, 2013 critically reviewed the existing literature with resepct to the presumptive role of aldehyde dehydrogenases (ALDH1, ALDH2 and ALDH3) in regulating HNE and other endogenous apoptogenic aldehydes (methional, malondialdehyde) showing conflicting results concerning the contribution of different ALDH isoforms to HNE detoxification [56].

ALDH2 may play an important role in neuronal injury, the overexpression or activation in ALDH2 deficient rats providing neuroprotection via enhanced HNE removal [57]. Supportive to this, the neuroprotective effect of protein kinase Cε—a positive regulator of ALDH2 was abolished in ALDH2-knockdown rats. Notably, ALDH2 also contributes to myocardial protection from ischaemia/reperfusion injury which appers to be attributable to HNE detoxification as well as autophagy regulated by AMPK- and Akt-mTOR signalling during ischaemia and reperfusion [58].

Furthermore, the ALDH3 subfamily comprises a group of enzymes which oxidize medium-chain aliphatic and aromatic aldehydes (e.g., fatty and peroxidic aldehydes). ALDH3A1, a phase II drug-metabolizing enzyme is poorly expressed in the normal liver but present at high levels in the lung, stomach, cornea and keratinocytes, cells with a high ALDH3A1 content revealing an increased resistance to the cytostatic and cytotoxic effects of lipidic aldehydes [59]. Transfection studies demonstrated that ALDH3, but not class 1 ALDH, protects from severe GSH depletion and HNE mediated apoptosis in V79 fibroblasts and RAW264.7 macrophages which is associated with reduced levels of HNE protein-adducts [60]. Moreover, HNE serves as substrate for ALDH3 catalyzed oxidation suggesting that the oxidative decomposition of HNE by class 3 ALDH constitutes an important mechanism protecting from HNE toxicity [60]. Notably, this report also showed that the contributions of HNE functional groups to HNE toxicity follows the order aldehyde ≥ C2=C3 double bond >> C4-hydroxyl group. The role of ALDH3A1 in oxidative stress responses was further characterized in a rabbit corneal keratocyte cell line (TRK43) overexpressing ALDH3A1 which confirmed the protective role of ALDH3A1 towards the adverse effects of HNE which may occur under conditions of oxidative stress [61]. Additionally, Yoval-Sanchez *et al.*, 2012 reported that human ALDHs reveal different sensitivities to inactivation by LPO-derived aldehydes, ALDH2 most effciently oxidizing HNE, acrolein, and MDA [62]. Interestingly, while these compounds are also processed by ALDH1A1, ALDH3A1 specifically oxidizes HNE. Of note, ALDH2 is irreversibly inactivated by these LPO-metabolites which may target the ALDH aldehyde and NAD^+^ binding sites. In contrast, ALDH3A1 tolerates concentrations of HNE or MDA of 20 mM. Hence, the pronounced resistance towards inactivation by aldehydic LPO metabolites emphasizes a particular role of ALDH3A1 (and also ALDH1A1) in cellular detoxification of LPO-derived aldehydes [62].

#### 3.2.4. Reduction

The opposite reaction, reduction of the carbonyl group, can be catalyzed by alcohol dehydrogenase (ADH) and NADH (Figure 4). Of special relevance, amyloid beta peptide (Aβ) binding-alcohol dehydrogenase (ABAD) which is present in the brain metabolizes different substrates including β-estradiol as well as n-isopropanol in addition to its Aβ binding property. In the pathogenesis of Alzheimer disease (AD), the association of mitochondria derived oxidative stress with Aβ plays a considerable role and the aldehyde detoxification by ABAD has been suspected to play a particular role. Indeed, in SH-SY5Y neuroblastoma cells, representing a widely used *in vitro* model of neuronal function and differentiation, ABAD is able to decompose HNE and to antagonize HNE cytotoxicity wich is suppressed by Aβ [63]. Based on this, it is suggested that the impairment of ABAD mediated HNE-detoxification by Aβ contributes to the pathogenesis of AD.

A second enzyme, *aldose reductase*, preferentially reduces the glutathione conjugate of HNE. Aldose reductase protects against early atherosclerotic lesion formation in apolipoprotein E-null mice by removing HNE [64] and protects also against myocardial ischemia-reperfusion injury in mice [65].

Several studies indicate that a third group of enzymes, namely several members of the *aldo-keto reductase* (AKR) family are also involved in the metabolism of HNE [66,67,68]. For instance, AKR1C3 contributes to HNE reduction and thereby protects human SH-SY5Y cells from HNE cytotoxicity. Notably, AKR expression is under the control of Nrf2, a regulatorys effect which may be crucial to the cytoprotection conferred by Nrf2 towards the toxic effects of lipid peroxides. Similarly, AKR7A2 renders V79-4 Chinese hamster lung cells resitant towards HNE cytotoxicity, but fails to have the same effect on the toxicity of crotonaldehyde and acrolein [66]. Overexpression of AKR7A2 in V79 cells lowered HNE genotoxicity, improved the resistance to the redox-cycler menadione by lowering menadione-induced ROS generation and GSH depletion. With resepct to this, it cannot be ruled out that the participation of AKRs like AKR7A2 in cellular detoxifying pathways may also be of relevance to antioxidant defenses *in vivo.* This is also indicated by the activity of AKR1B10, another AKR isoform which detoxifies HNE and other dietary and lipid-derived α,β-unsaturated carbonyls at physiological levels [69]. In addition, retinals have been shown to be the best substrate of AKR1B10 [70]. Recombinant rat AKR1B10 (R1B10), derived from the corresponding cDNA isolated from rat brain reduces different aldehydes including HHE, HNE and 4-oxo-2-nonenal as well as α-dicarbonyls such as methylglyoxal and 3-deoxyglucosone, NADPH serving as preferred coenzyme [71]. 4-oxo-2-nonenal is most efficiently reduced into 4-oxo-2-nonenol by recombinant R1B10 which also lowers its cytotoxic properties. Expression of R1B10 (mRNA) is highest in rat brain and heart, low levels detectable also in other tissues and skin fibroblasts. Similar to AKR7A2, these findings suggest that R1B10 serves antioxidant defenses in rat tissues which also holds true for rat AKR1C15, which is upregulated by HNE and protects endothelial cells from HNE mediated cell damage [72].

In photoreceptor cells a fourth group of HNE reducing enzymes was detected: the *retinol dehydrogenases* RDH11 and RDH12, which are capable of reducing LPO products such as HNE. As demonstrated by Marchette *et al.* [73], expression of RDH11 and RDH12 in human embryonic kidney (HEK-293) cells suppressed HNE protein modification and apoptosis triggered by this aldehyde. Moreover, LPO and HNE formation is induced upon exposure of the mouse retina to bright light peroxidation which is accompanied by HNE protein modification in the inner segments of the photoreceptor containing RDH11 and RDH12. Interestingly, HNE adduct formation and light-induced photoreceptor apoptosis was suppressed only by RDH12 which suggests a substrate specifity of RDH12 for HNE. From the mechanistical aspect, the protection from the light-induced prooxidant and cytotoxic effects is considered to be based on the reduction of HNE by RDH12 to the nontoxic alcohol 1,4-dihydoxy-nonene. It is worth to note that the mutation of the RDH12 gene is causal to *early onset retinal dystrophy Leber congenital amaurosis* (LCA), a severe inherited eye-disease with a still incompletely understood pathogenesis.

Finally, the carbonyl group of HNE can also be reduced or oxidized by members of the *cytochrome P450* family. For example, the incubation of cytochrome P450 3A4 with HNE, O_2_, and NADPH leads to the formation of several compounds including 1,4-dihydroxynonene (DHN) and 4-hydroxy-2-nonenoic acid (HNA) [74]. The reduction reaction is catalyzed also by other P450 isoforms especially (human) P450 2B6, revealing an activity similar to P450 3A4 and P450 1A2, P450 2J2, (mouse) P450 2c29, which are less active. In contrast, human P450 2E1 and rabbit P450 2B4 lack a reducing activity of the carbonyl group.

### 3.3. Reactions of the Hydroxy Group

The formation of cyclic hemi-acetals—as it is known for sugars—as secondary reactions has already been mentioned above. Similarly, the oxidation of HNE to 4-oxo-nonenal (Figure 4) has also been described as a secondary event only.

In addition, catabolic HNE degradation in the liver may also involve *phosphorylation* of the hydroxy group [75] as well as ω*-oxidation*, *i.e.*, the oxidation of the methyl group. ω-Oxidation, following oxidation of the carbonyl group is indicated by the presence of intermediates (4-hydroxynonanedioyl-CoA, 4-hydroxyheptanedioyl-CoA and 4-hydroxy-pentanedioyl-CoA) which account for a degradation of HNE from either end of the molecule [76].

## 4. Biophysical Effects

Besides biochemical effects, also biophysical changes of protein and lipid membrane conformation—e.g., loss of phospholipid asymmetry have to be taken into consideration. Using electron paramagnetic resonance spectroscopy in combination with protein-specific spin labeling (2,2,6,6-tetramethyl-4-maleimidopiperidin-1-oxyl), the induction of conformational changes of synaptosomal membrane proteins by physiologically relevant HNE concentrations (1 µM) was demonstrated [77].

HNE may also interfere with membrane fluidity, albeit results dealing with this aspect are conflicting. In gerbil synaptosomal membranes, fluidity of the bilayer is increased at higher HNE conentrations (*i.e.*, 50 µM) [77]. On the contrary, the fluidity of hepatic mitochondrial membranes in rats was decreased by HNE and to a lower extent also MDA supposedly as result of a direct interaction of HNE with membrane phospholipids [78]. Also, HNE directly inhibits the dopamine transporter and also decreases Na^+^/K^+^ ATPase activity in synaptosomes, the effect on Na^+^/K^+^ ATPase activity most likely resulting from HNE induced loss of membrane fluidity [79].

## 5. Biochemical Targets of HNE

The cellular reactions to HNE are strongly dose-dependent. According to recent concepts and supported by transcriptome analysis, low HNE concentrations provide protection from oxidative-stress mediated cell damage while increasing HNE doses promote genotoxic effects and initiate apoptosis or severely deregulates cell integrity [80].

The smallest reported biochemical target of HNE is hydrogen sulfide (H_2_S) which has been recognized as neuromodulator and gasotransmitter. H_2_S protects SH-SY5Y neuroblastoma cells from HNE cytotoxicity and HNE-related protein modification leading to the assumption that H_2_S could represent an important antagonist to “carbonyl stress” arising from aldehydic LPO metabolites [81].

### 5.1. Reactions with Peptides and Proteins

From a quantitative perspective, proteins and peptides represent the most important class of biomolecules which are targeted by HNE. About 1% to 8% of the HNE formed in a cell are considered to modify proteins [82] and several investigations have been carried out to identify protein targets of HNE modification [83,84,85]. For example, more than 1500 proteins were identified as HNE targets in human colon carcinoma cells, 417 of these proteins showing a significant concentration dependent HNE-adduct formation [86]. Another study by Chavez *et al.* [85] using HNE treated human monocytic THP-1 cells demonstrated distinct, site-specific (His, Cys) HNE modifications of 16 proteins comprising cytoskeletal elements (tubulin α-1B chain, α-actinin-4, vimentin), proteins associated with stress responses and metabolic enzymes (D-3-phosphoglycerate dehydrogenase, aldolase A). Also, heat shock protein Hsp90 as well as the endoplasmic stress response protein, protein disulfide isomerase (PDI) may also become targets of HNE generated by alcohol-induced oxidative stress in rat liver [87]. Another illustrative *in vivo* example of selective targeting of enzymes involved in glycolysis, energy production as well as CO_2_ hydration has been described for the ventilatory muscles of rats under the conditions of severe sepsis and strenuous muscle contractions [88]. Under these conditions, HNE adduction of enolase 3b, aldolase and triosephosphate isomerase 1, creatine kinase, carbonic anhydrase III, aconitase 2, dihydrolipoamide dehydrogenase, and electron transfer flavoprotein-beta is detectable. Together these studies account for different sensitivities to HNE protein modification between cellular proteins. In this context it has to be noted that HNE protein adduct formation affects the whole cell while prooxidant regimens such as H_2_O_2_ preferentially trigger cytosolic protein oxidation [89].

Enolase activity is incrementally declining at increasing HNE concentrations *in vitro* which demonstrates a dose-dependent negative effect of HNE-adduct formation on enzyme activity [88]. Indeed, in most instances protein function is impaired by HNE adduct formation. The aforementioned modification of Hsp70 and Hsp90 in alcoholic liver disease adversely interferes with the protein binding and ATPase activity of these stress response proteins. Moreover, HNE adducts negatively affect the activity of *protein disulfide isomerase* which is required for the proper establishment of disulfide bonds in protein folding. As a consequence, HNE modification will promote the accumulation of damaged or misfolded proteins which is detectable in the liver upon chronic alcohol ingestion [87,90,91].

It is well established that HNE forms adducts with three different side chains in proteins, namely Cys, His and Lys. The differential reactivity of these residues as well as of Arg and Glu for adduct formation with HNE in synthetic poly-aminoacid model compounds has been studied [92]. It turned out that Cys residues displayed by far the highest reactivity; the order of the molar HNE/AA ratio was Cys (0.6) >> His (1 × 10^−3^) > Lys (3 × 10^−4^) >> Arg (4 × 10^−5^). No reaction of HNE was detected with Glu. Similar results were obtained for the keto-derivative of HNE, 4-oxononenal: (Cys >> His > Lys > Arg) with Arg being a target for 4-oxononenal but generally not for HNE [93]. However, one case has been described (cytochrome c), where Arg^38^ is also modified by HNE besides His^33^ and Lys^87^ residues [94]. The above mentioned order does not imply that Cys residues are always the preferential targets of HNE in proteins. Several other factors besides reactivity may determine the adduction site, such as polarity of the microenvironment and accessibility in the tertiary/quarternary structure. For example it has been shown for HNE- and 4-oxo-2-nonenal-treated myoglobin and apomyoglobin, that the latter more “open” protein structure resulted in more extensive modification [95]. Changes in the polarity of the microenvironment may explain, why the presence of Arg in a model peptide increases the reaction rate of Cys for adduct formation with HNE as well as 4-oxononenal by a factor of 5–6 compared to the Cys nucleophile alone [93]. The impact of accessibility and acidity has been shown for actin. Among several Cys residues only Cys^374^ is adducted by HNE because of significant accessible surface and substantial thiol acidity due to the particular microenvironment surrounding Cys^374^ [96].

Furthermore, the presence of other ligands bound to proteins can strongly influence adduct formation with HNE. The *in vitro* modification of Cys^120^ of epithelial fatty acid-binding protein by HNE is markedly potentiated by noncovalently bound fatty acids [97].

Adduct formation of HNE with proteins is a rather fast reaction. The half life for the covalent modification of the epithelial fatty acid-binding protein was reported to be t_1/2_ < 60 s *in vitro* [97]. Adduct formation of HNE with protein thiols can be reversible in the presence of low molecular thiols such as GSH. Physiological (e.g., 4 mM) concentrations of GSH were capable of removing the HNE adducts from PDI [98]. It is likely this mechanism serves as a protective mechanism against inactivation by HNE and other lipid peroxidation products.

Protein adducts of HNE are physiological constituents of mammalian organisms. Notably these adducts occur in blood, where they increase with age [99]. In an age-dependence study on HNE-adducted serum proteins in Fischer 344 rats albumin, transferrin and immunoglobulins were most prominently adducted by HNE [100]. In a similar study HNE-adducted proteins have also been determined in serum of aging Fischer 344 rats [101]. Among these, 16 are involved in blood coagulation, lipid transport, blood pressure regulation, and protease inhibition.

Recent evidence suggesting a role for protein modification by HNE in the pathogenesis of several diseases has been reviewed in [102]. The precise mechanisms are currently unknown but likely result from adduction of proteins involved in cellular homeostasis or biological signaling. The extent of HNE adduction to proteins can increase dramatically under pathophysiological conditions. e.g., using a heterotropic rat heart transplantation model the formation of protein adducts of HNE was studied as a response to cold storage and warm blood reperfusion in the recipient [103]. In hearts submitted to ischemia only, the adduct formation was low (<1% of the tissue area). However, transplantation and reperfusion in the recipient increased the amount of protein adducts to about 6% and it was assumed that this damage negatively affects longterm survival of the transplant.

In a murine model for alcoholic liver disease 414 protein targets for modification by reactive aldehydes (HNE, 4-oxononenal, acrolein, and MDA) were identified by a hydrazide method coupled to a highly sensitive 2-dimensional liquid chromatography tandem mass spectrometry (2D LC-MS/MS or MuDPIT) technique [104].

Also for individual proteins a high degree of HNE adduction has been reported in a condition of oxidative stress. In progressive supranuclear palsy, a neurodegenerative disorder, which may possibly be induced by oxidative stress, two-thirds of total glutathione peroxidase in the cerebrospinal fluid was found to be adducted with HNE. It was assumed that the antioxidant system might be unable to function effectively because of conjugation with HNE [105].

Adduction by HNE tends to make the targeted protein more succeptible to degradation by the proteasomal pathway which is responsible for most intracellular proteolysis. While under normal conditions the proteasomal system is able to remove the bulk of oxidatively damaged and modified proteins, under severe oxidative stress the accumulation of modified proteins occurs. This takes place either due to cross-linking of proteins or due to malfunctioning of the proteolytic machinery of the cell. The efficiency of the removal of HNE-modified proteins clearly depends on the dose of cellular exposure to HNE. Taking into consideration the permanent formation of HNE and its metabolism it has been shown that low levels of HNE-modification are sufficient to increase the proteolytic susceptibility of proteins [106]. Equine ADH treated with a 2-fold molar excess of HNE was degraded by a rabbit reticulocyte lysate system approximately 1.5-fold faster than the control, while treatment with concentrations up to 100-fold molar excess of HNE were inhibitory to degradation [107].

On the other hand, the proteasome itself is a target for HNE adduction causing inhibition of proteolysis [108,109]. In the liver of rats which were fed ethanol for one month HNE—produced presumably by P450 cytochrome 2E1 (CYP2E1)—formed an adduct with the Rpt4 subunit of the 26 S proteasome [110]. It was proposed that this adduct could impede the association of 19 S and 20 S subunits and thus accounts for the observed decrease of proteasomal activity. In accordance with these data a decline in trypsin-like (but not chymotrypsin-like) activity of the proteasome in HepG2 cells overexpressing CYP2E1 was associated with higher levels of HNE-modified proteins compared to control cells [111]. An altered balance between protein modification, ubiquitination, and degradation was proposed for these cells.

Modification of another subunit of the proteasome has also been reported leading to a decline of chymotrypsin-like activity. A catalytic site-specific inhibition of the α6/C2 subunit of the 20 S proteasome by HNE was observed in liver [112]. Furthermore, the degradation of proteins by a proteasome-independent pathway may also be triggered by adduct formation with HNE as was shown for glyceraldehyde-3-phosphate dehydrogenase in U937 cells [113].

The reaction of HNE with proteins is frequently associated with their covalent crosslinking leading to the formation of fluorophores. A major fluorophore has been identified as a lysine-derived dihydro-pyrrole derivative [114]. Finally, it should be mentioned that protein-HNE adducts also represent useful biomarkers of oxidative stress, lipid peroxidation and oxidative homeostasis [115].

In the next chapter several well studied examples of peptides and proteins are described as targets of HNE including synaptic proteins such as synaptosomal-associated protein 25 [116].

#### 5.1.1. Substrates

##### 5.1.1.1. Glutathione

Glutathione (GSH), whose concentration in mammalian liver is in the millimolar range, is likely to be the quantitatively most important target of HNE. Several glutathione-S-transferases (GST) such as GSTA4-4 and hGST5.8 are involved in the conjugation of HNE to GSH in humans. The isoenzyme GSTA4-4 with high specificity for HNE has been found in human liver mitochondria. The specific mitochondrial GST activities toward HNE exceeded that observed in liver cytosol. These observations are suggestive of a role of GST in protecting against mitochondrial injury during the secondary phase of oxidative stress, or modulation of HNE-mediated mitochondrial signaling pathways [117].

The conjugation of HNE with glutathione is reversible. Both the spontaneous and the GST-catalyzed retro-Michael cleavages of HNE-GSH are the source of HNE in the urine of rats [118]. Strategies such as irrigation with gamma-glutamyl-cysteine to increase glutathione levels have been suggested as effective means for normalizing pathophysiological HNE levels after traumatic injuries [119].

GSTs (in humans hGSTA4-4 and hGST5.8) and the transporter 76 kDa Ral-binding GTPase activating protein (RLIP76) regulate intracellular concentrations of HNE through a coordinated action in various human cell lines and erythrocytes and catalyzes the ATP-dependent transport of the conjugate into the extracellular space, *i.e.*, a mild stress caused by heat, UV-A, or hydrogen peroxide with no apparent effect on the cells in culture caused a rapid, transient induction of hGST5.8 and RLIP76. These stress preconditioned cells acquired the ability to metabolize and exclude HNE at an accelerated pace and acquired relative resistance to apoptosis by UV and oxidative stress as compared to unconditioned control cells [120].

Patrick *et al.*, 2005 demonstrated that depletion of HNE in hGSTA4-4-transfected adherent HLE B-3 cells results in profound changes in gene expression, phenotypic transformation and immortalization [121]. At the genetic level this transfection caused changes in the expression of genes involved in cell adhesion, cell cycle control, proliferation, cell growth, and apoptosis, which is consistent with the phenotypic changes of the transformed cells. For example, the expression of p53, p21, p16, fibronectin 1, laminin gamma 1, connexin 43, Fas, integrinα6, TGFα, and c-jun was down-regulated, while the expression of protein kinase C beta II, c-myc, cyclin-dependent kinase 2, extracellular signal regulated kinase and transforming growth factor β1 was up-regulated [121].

The consequences of severe oxidative stress have also been studied in GSTA4-4 deficient mice. The acute hepatotoxicity of CCl_4_ in GSTA4-4^−/−^ mice was compared with wild type GSTA4-4^+/+^ mice [122]. The results indicated that GSTA4-4 is an important component during the early stages (1–6 h) of cellular defense against oxidative stress and lipid peroxidation, although it is not effective in protecting against the degree of overall cell injury.

In addition chronic hepatotoxicity was studied in mGSTA4 null mice. Mice homozygous for the disrupted mGSTA4 allele were viable and appeared normal except for lower litter size, higher fat content in bones, and greater susceptibility to bacterial infection. The null mice had a lower survival time than wild-type controls when chronically treated with relatively low doses of paraquat. These mice had a reduced ability to conjugate HNE, and had an increased steady-state level of HNE in tissues [123].

The genotoxicity of HNE is highly dependent on cellular GSH status and those GSTs that contribute toward HNE conjugation, including hGSTA4-4. Depletion of GSH in HT29 cells, derived from a human colorectal adenocarcinoma, by BSO (l-buthionine-S,R-sulfoximine) decreased cellular GSH levels by 77% without significant changes in cell viability. Associated with this decrease was a 2-fold higher level of HNE-induced DNA damage as measured by the comet assay [124].

The glutathione adduct of HNE has signaling properties of its own [125]. For instance, it enhances the peritoneal leukocyte infiltration and stimulates the formation of proinflammatory lipid mediators. Moreover, the reduced form of the glutathione conjugate of HNE (GS-DHN) elicits strong mitogenic signaling in smooth muscle cells. Also, GSH-HNE is not only a product of adipocyte oxidative stress but also an activator of macrophage inflammation [126] and is pro-inflammatory *in vivo* in mice [127].

##### 5.1.1.2. Carnosine

Carnosine is a dipeptide (beta-alanyl-l-histidine) present in mammalian tissue and in particular at high concentration in skeletal muscle. Growing evidence indicates that it acts as quencher of reactive and cytotoxic carbonyl species including HNE [45,128]. The reaction mechanism was found to be an intra-molecular Michael addition. Two reaction products of carnosine were identified, in a pH-dependent equilibrium: (a) the Michael adduct, stabilized as a 5-member cyclic hemi-acetal and (b) an imine macrocyclic derivative. The adduction of carnosine to HNE thus appears to start with the formation of a reversible α,β-unsaturated imine, followed by ring closure through an intra-molecular Michael addition [128]. By trapping HNE in stable covalent adducts, carnosine can inhibit HNE-induced protein cross-linking*.*

The biological role of carnosine as a quencher of α,β-unsaturated aldehydes was verified by detecting carnosine-HNE adducts in oxidized rat skeletal muscle homogenates [129]. In a therapeutic approach it was shown that dietary carnosine prevents early atherosclerotic lesion formation in apolipoprotein E-null mice [130]. The facile Michael adduct formation of carnosine with 4-hydroxyalkenal species was explored and a sensitive, facile, shotgun lipidomics-based method was developed for quantification of these compounds directly from organic solvent lipid extracts of biological samples [131].

A perspective for the design of novel derivatives, active as exogenous agents able to detoxify carbonyl compounds has been proposed [132]. Histidyl-containing carnosine analogues bearing hydrazide or 1,2-diol moieties have been synthesized, some of which have demonstrated higher aldehyde-sequestering efficiency than carnosine and were also efficient in protecting SH-SY5Y neuroblastoma cells and rat hippocampal neurons from HNE-mediated death. The cytoprotective efficacy of these compounds suggests their potential use as therapeutic agents for disorders that involve excessive membrane lipid peroxidation and HNE-mediated neuronal toxicity [133]. Vistoli *et al.*, 2009 described a set of aryl derivatives that are characterized by high stability in human plasma and a quenching activity toward HNE, as a model of reactive carbonyl species, up to threefold greater than d-carnosine [134].

The synthesis and the physicochemical and biological characterization of a series of carnosine amides bearing alkyl substituents on the amido group endowed with different lipophilicity have been described by Bertinaria *et al.*, 2011 [135]. These products can be considered as metabolically stable analogues of carnosine and are worth of additional investigation as potential neuroprotective agents.

##### 5.1.1.3. Thioredoxin

Thioredoxins (TRXs) are scavengers of intracellular ROS and participate in the anti-oxidant system of the retina. Disruption of these systems leads to dysfunction of retinal pigment epithelial (RPE) cells, which then accelerates the development of age-related macular degeneration (AMD). The role of TRXs in the protection against HNE-induced oxidative stress was studied in RPE cells by assessing the effect of TRX overexpression on cell viability, morphology, NFκB expression, and mitochondrial membrane potential [136]. It was found that overexpression of TRXs reduced cell death caused by HNE: HNE caused perinuclear NFκB accumulation, which was absent in TRX-overexpressing cells. Moreover, overexpression of TRXs prevented depolarization of mitochondrial membranes. TRX2 was more effective than TRX1 in maintaining the membrane potential. The difference in the protective effects of these TRXs against oxidative stress may be due to their expression profile. TRX2 was expressed in the mitochondria, while TRX1 was expressed in the cytoplasm. It was therefore suggested that the effect of TRXs on mitochondria may be a key to prevent oxidative stress in RPE cells [137].

##### 5.1.1.4. Cytochrome c

The nature of cytochrome c modification by HNE was investigated in an *in vitro* study [138]. The overwhelming reaction observed is Michael addition by Lys side-chains in addition to the modification of His^33^. While the Lys-HNE adducts were generally observed to be reversible, the HNE-His^33^ was found to be stable with half of the formed adduct surviving the denaturation and proteolysis protocols used to generate proteolytic peptides for LC-ESI-MS/MS.

#### 5.1.2. Enzymes

When the amino acid targets of HNE are in the active site of an enzyme, the activity is frequently diminished. It was therefore proposed that HNE for example may disrupt the active site of lipoxygenase-1 by forming a Michael adduct with one or more of the three histidines that ligate the iron active site [139]. However, the inactivation of enzymes by HNE need not necessarily be due to the modification of the catalytic center but may be due to the selective modification of amino acids primarily located on the surface of the enzyme as has been shown for glyceraldehyde-3-phosphate dehydrogenase (GAPDH). HNE is a potent inhibitor of sulfhydryl enzymes such as the glycolytic enzyme GAPDH. It has therefore been suggested that HNE exerts an inhibitory effect on the enzyme due to the modification of Cys^149^ at the catalytic site generating the HNE-cysteine Michael addition-type adduct [140]. To test this hypothesis, ESI-MS of tryptic peptides was carried out and identified five peptides, which contained the HNE adducts at His^164^, Cys^244^, Cys^281^, His^327^, and Lys^331^ and revealed that both His^164^ and Cys^281^ were very rapidly modified within 5 min, followed by Cys^244^ at 15 min and His^327^ and Lys^331^ at 30 min. However, a modification of the catalytic center, Cys^149^, by HNE was not observed [141].

As mentioned, the inhibition of enzymes is in some cases reversible in the presence of thiol-containing compounds such as glutathione [142,143] or acetylcysteine. For example, cysteine restores the activity of the pyruvate dehydrogenase complex [144] but in most instances irreversible destruction takes place which may also be site-specific [145]. Profound consequences are to be expected when HNE forms adducts both with the substrate and the enzyme of a biochemical reaction as is the case of the TRX/TrxR system. TrxR is most sensitive to inhibition by HNE. This seleno-enzyme regulates redox-sensitive proteins involved in inflammation and carcinogenesis, including ribonucleotide reductase, p53, NFκB, and others. HNE and also its precursor, the 15-lipoxygenase-1 product, 15(S)-hydroperoxy-5,8,11-*cis*-13-*trans*-eicosatetraenoic acid, inhibit TrxR with IC_50_ = 1 µM and 13 µM, respectively, in colo-rectal cancer cells [146]. Besides inhibition of TrxR irreversible inactivation of TRX occurs. A pair of Cys residues (Cys^32^/Cys^35^) in Trx and the selenocysteine (Sec) and Cys residues (Cys^496^/Sec^497^) in the active site of TrxR are primary targets for HNE modification [147]. Because the TRX system is one of the core regulatory enzymes of cellular functions, it is assumed that inhibition of both TrxR and TRX by HNE provides a possibly novel mechanism for the explanation of its cytotoxic effect and signaling activity, as well as the further damage indirectly caused under oxidative stress conditions.

However, HNE is also an inducer of TrxR [148]. It has been shown to exert an adaptive cytoprotective effect at low concentrations through induction of TrxR 1 via transcriptional activation of Nrf2. As a consequence pretreatment with HNE at sublethal concentrations significantly protected PC12 cells rat adrenal pheochromocytoma line, against the subsequent oxidative cell death induced by H_2_O_2_ and 6-hydroxydopamine. Since HNE also exhibited adaptive protection in human arterial endothelial cells far reaching conclusions were drawn from these findings. It was suggested that this may be a general effect of HNE and may lead to a reappraisal of the eventual role of ROS and LPO in organisms [149]. It has to be determined whether these complex effects of HNE—inhibitor on the one side, inducer on the other—can be explained by tissue-specific responses.

In a few instances activation rather than inhibition of enzymes may occur. Interestingly, in the case of a protease HNE is promoting reactions via four separate mechanisms under pathophysiological conditions: activation and induction of the enzyme, modification of the substrate and inhibition of expression of the substrate [150]. During the destruction of cartilage HNE is prominently produced in osteoarthritic (OA) synovial cells. *In vitro*, HNE binding to matrix metalloproteinase 13 (MMP-13) activated this enzyme, and modification of type II collagen (Col II) by HNE accelerated its degradation by MMP-13. The authors concluded that the increased level of HNE in OA cartilage and the ability of HNE to induce transcriptional and posttranslational modifications of Col II and MMP-13 suggest that HNE could play a role in OA [150].

In the following section several examples of enzymes from different enzyme classes and other proteins with pathophysiological significance are presented as targets of HNE. An overview over the enzymes identified to form adducts with HNE has been presented previously [4].

##### 5.1.2.1. Oxidoreductases

###### 5.1.2.1.1. Lactate Dehydrogenase

Lactate dehydrogenase (LDH) activity can regenerate NADH. Mass spectrometric examination revealed that HNE binds with cysteine and histidine residues of LDH at pH 5.6 and 7.4, and covalent binding of HNE decreases NADH formation [151].

###### 5.1.2.1.2. Glyceraldehyde-3-Phosphate Dehydrogenase (GAPDH)

Schlisser *et al.*, 2009 conducted an investigation on organogenesis in mice with the goal to determine whether exposure of CD1 mice to the model teratogen hydroxyurea (HU) on gestation day nine generates region-specific HNE-protein adducts in the embryo, and to identify the proteins targeted [152]. They found out that the formation of HNE-protein adducts was elevated in the caudal region of control embryos and that HU exposure further increased HNE-protein adduct formation in this area. Interestingly, among the eight identified HNE-modified proteins, GAPDH appears to play a specific role since it was shown that HNE adducts reduced GAPDH enzymatic activity by 20%. The authors therefore concluded that GAPDH may play a role in stress response during development.

##### 5.1.2.2. Transferases

###### 5.1.2.2.1. Glutathione-S-Transferase (GST)

HNE is not only a substrate for GSTs, but also a modifier under pathophysiological conditions such as Alzheimer’s disease (AD), a neurodegenerative disorder characterized pathologically by intracellular inclusions including neurofibrillary tangles and senile plaques. Oxidative stress is associated with the pathogenesis of the disease leading to oxidative modifications of cellular macromulecules such as lipids and proteins [153,154], and an increased formation of HNE [155]. Since the alpha class of GST can detoxify HNE and may interact with the multidrug resistance protein-1 (MRP1) to export the glutathione conjugate of HNE to confer cellular protection. Sultana and Butterfield, 2004 investigated oxidative modifications of GST and MRP1 in AD brain [156]. They found that HNE is covalently bound to GST and MRP1 proteins in excess in AD brain and suggested that HNE may be an important mediator of oxidative stress-induced impairment of this detoxifying system which thus may play a role in promoting neuronal cell death.

Besides modification HNE can induce the expression of GST via Nrf2. Nrf2 regulates a battery of antioxidative and phase II drug metabolizing/detoxifying genes via binding to the antioxidant response elements (ARE). Nrf2-ARE signaling plays a central role in protecting cells from a wide spectrum of reactive toxic species including reactive oxygen/nitrogen species (RONS). The role of Nrf2 in regulating the HNE induced gene expression of antioxidant and detoxifying enzymes was studied in HeLa cells [157]. When HeLa cells were treated with HNE, Nrf2 rapidly translocated into the nucleus and increased Nrf2 protein in the nuclear fraction causing a dose-dependent transcriptional activity of ARE followed by the expression of GST A4, AKR1C1 and heme oxygenase-1 (HO-1). Moreover, this induction was attenuated by knocking down Nrf2, highlighting the role of Nrf2 in mediating HNE induced expression of antioxidant and detoxifying genes.

GSTA4-4 has been postulated to be a rigid template that is preorganized for HNE metabolism. However, the combination of high substrate chemoselectivity and low substrate stereoselectivity is intriguing [158]. GSTA4-4 must metabolize both enantiomers of HNE to efficiently detoxify the biologically formed mixture and achieves this by an ideal location of the active site residue Arg15 enabling the interaction with the 4-hydroxyl group of either HNE enantiomer [158].

###### 5.1.2.2.2. Liver Kinase B1 (LKB1)

LKB1 is characterized as a serine/threonine kinase that phosphorylates and activates the AMP-activated protein kinase (AMPK) and 12 other AMPK-related kinases. HNE treatment (10 µM for 1 h) of human embryonic kidney 293T (HEK293T) cells expressing LKB1 resulted in the formation of HNE-LKB1 adducts and decreased LKB1 kinase activity but had no effect on the association of LKB1 with its adaptor proteins sterile-20-related adaptor and mouse protein 25. Mutation of LKB1 Lys^97^ residue reduced HNE adduct formation and attenuated the effect of HNE on LKB1 activity. It was therefore concluded that adduction of LKB1 Lys^97^ mediates the inhibitory effect of HNE [159].

###### 5.1.2.2.3. 5'-AMP-Activated Protein Kinase (AMPK)

AMPK is a major regulator of β-oxidation, which is altered in ALD. In an *in vitro* cellular model, AMPK was identified as a direct target of HNE adduction resulting in inhibition of H_2_O_2_- and 5-aminoimidazole-4-carboxyamide ribonucleoside (AICAR)-induced downstream signaling [160]. Using a murine model of ALD the authors were able to demonstrate that treatment with high concentrations of ethanol resulted in an increase in phosphorylated as well as carbonylated AMPKα. Mass spectrometry thereafter identified Michael addition adducts of HNE on Cys^130^, Cys^174^, Cys^227^, and Cys^304^ on rAMPKα and Cys^225^ on rAMPKβ which by molecular modeling analysis of the adducted sites revealed an inhibition of AMPK by steric hindrance of the active site pocket and inhibition of hydrogen peroxide induced oxidation.

###### 5.1.2.2.4. ZAK Kinase (Sterile Alpha Motif and Leucine Zipper Containing Kinase AZK)

ZAK kinase is a member of the MAPKKK family of signal transduction molecules. It contains an N-terminal kinase catalytic domain, followed by a leucine zipper motif and a sterile-alpha motif (SAM). It mediates gamma radiation signaling leading to cell cycle arrest and the activity of this protein plays a role in cell cycle checkpoint regulation. The protein also has pro-apoptotic activity.

ZAK kinase is adducted by HNE on a conserved, active site-proximal cysteine and the resulting enzyme inhibition generates a negative feedback mechanism that can suppress the activation of JNK pathways normally induced by oxidative stress [161].

###### 5.1.2.2.5. Serine/Threonine-Protein Kinase AKT2 (Proteinkinase B2)

The effects of HNE on insulin-dependent stimulation of the AKT2 pathway have been evaluated by Shearn *et al.*, 2011 [162]. The regulatory effect of AKT2 is mediated through serine and/or threonine phosphorylation of a range of downstream substrates. Following HNE treatment, the level of downstream phosphorylation of Akt substrates such as glycogen synthase kinase-3-β (GSK3β) was significantly decreased in the hepatocellular carcinoma cell line HepG2, and this effect was shown to be mediated by Michael addition adducts of HNE with His^196^ and Cys^311^ of rAkt2 suggesting inhibition of GSK3β peptide binding in the Akt2 substrate binding pocket*.* This inhibition of Akt by HNE provides a novel mechanism for increased insulin resistance in ALD [162].

##### 5.1.2.3. Hydrolases

###### 5.1.2.3.1. ATP Synthase

Oxidative stress has been proposed as a mechanism for impaired beta-cell function in type 2 diabetes. Pancreatic islets from humans with type 2 diabetes were used to study the occurrence of HNE adducts in these cells [163]. The major HNE adduct was a 52-kDa protein seen with four different antibodies (two antibodies against HNE and two other antibodies generated against reactive small aliphatic compounds) that was also seen in islets of nondiabetic humans, rat islets, and insulinoma cells and in mitochondria of various rat tissues. It was identified as the β-chain of the mitochondrial F-ATP synthase, an enzyme responsible for 95% of ATP formed in tissues. The ATP synthase β-subunit has also been identified as a major target in isolated rat liver mitochondria upon HNE exposure [164].

###### 5.1.2.3.2. Phosphatase and Tensin Homolog Deleted on Chromosome 10 (PTEN)

The tumor suppressor PTEN is a key regulator of Akt or protein kinase B (Akt/PKB) activation in hepatocytes, and plays a role in the etiology of alcoholic liver disease. PTEN negatively regulates AKT activation via its lipid phosphatase activity. PTEN is a phosphatidylinositol 3-phosphatase catalyzing the removal of the 3-position phosphate from phosphatidylinositol-3,4,5-trisphosphate [PtdIns(3,4,5)P-3] to produce phosphatidylinositol-4,5-bisphosphate.

Treatment of both the human hepatocellular carcinoma cell line (HepG2) and primary hepatocytes with subcytotoxic concentrations of HNE resulted in the activation of Akt within 30 min as demonstrated by increased phosphorylation of residues Ser^473^ and Thr^308^ [165]. This increased phosphorylation is accompanied by a 6-fold increase in total PtdIns(3,4,5)P-3 and an increased immunostaining at the plasma membrane after HNE treatment. However, PTEN lipid phosphatase activity is decreased due to the formation of a single HNE adduct with the active site cysteine in PTEN (Cys^124^).

PTEN has also been used to demonstrate a new methodology to interrogate effects of reactive electrophiles on specific target proteins in cells [166]. In this study a target specific electrophile delivery platform was introduced that ultimately paves the way to interrogate effects of reactive electrophiles on specific target proteins in cells. This new methodology was demonstrated by photoinducible targeted delivery of HNE to the PTEN and Keap1. Covalent conjugation of the HNE-precursor to HaloTag fused to the target proteins enables directed HNE delivery upon photoactivation and allows a more precise determination of the pathophysiological consequences of HNE-induced protein modifications [166]. HaloTag again was designed as a modular protein tagging system that allows different functionalities to be linked onto a single genetic fusion, either in solution, in living cells, or in chemically fixed cells [167]. The protein tag (HaloTag) is a modified haloalkane dehalogenase designed to covalently bind to synthetic ligands (HaloTag ligands). The synthetic ligands comprise a chloroalkane linker attached to a variety of useful molecules, such as fluorescent dyes, affinity handles, or solid surfaces. Covalent bond formation between the protein tag and the chloroalkane linker is highly specific, occurs rapidly under physiological conditions, and is essentially irreversible. The utility of this system for cellular imaging and protein immobilization was demonstrated by analyzing multiple molecular processes associated with NFκB-mediated cellular physiology, including imaging of subcellular protein translocation and capture of protein-protein and protein-DNA complexes [167].

###### 5.1.2.3.3. Sirtuin 3 (SIRT3)

SIRT3 is a mitochondrial class III histone deacetylase, which is inhibited by HNE via a thiol-specific modification [168]. HNE covalently modifies *rSIRT3 at Cys^280^* altering the conformation of the zinc-binding domain and thus allosterically inhibits SIRT3 activity.

###### 5.1.2.3.4. Cathepsins

Since reduced lysosomal capacity may contribute to lipofuscinogenesis and progressive dysfunction of the retinal pigment epithelium (RPE) during the pathogenesis of age-related macular degeneration Krohne *et al.*, 2010 investigated the effects of HNE and MDA on both isolated lysosomes from primary human retinal pigment epithelial cells (RPE) and on cultured RPE cells. Both HNE and MDA inhibited the cysteine proteases cathepsin B and L at a concentration of 1 µM by 88%–94% and this effect was due to HNE and MDA adducts in the active center of these proteases [169].

###### 5.1.2.3.5. Neprilysin (NEP)

As one of the dominant amyloid-β peptide (Aβ) proteases, NEP plays a crucial role in maintaining a physiological balance between Aβ production and catabolism. Wang *et al.*, 2003 and 2009 showed that NEP is modified by HNE adducts, resulting in decreased activity in the brain of AD patients and cultured SH-SY5Y and H4 APP695wt cells [170,171]. The inactivation of NEP by HNE-adduction was associated with, at least partly, reduced Aβ cleavage and enhanced Aβ accumulation [171]. The HNE-induced modification and inactivation can be prevented by the carbonyl-scavenger *N*-acetylcysteine [172].

##### 5.1.2.4. Lyases

###### 5.1.2.4.1. Mitochondrial Aconitase (ACO2)

ACO2 is inactivated by HNE. Liu *et al.*, 2013 related the inactivation of ACO2 by HNE to structural features and determined the HNE addition reaction rates using the iTRAQ approach (isobaric Tags for Relative and Absolute Quantitation) [173]. The most reactive sites were Cys^358^, Cys^421^, and Cys^424^, the three iron-sulfur cluster-coordinating cysteines, Cys^99^, the closest non-ligated cysteine to the cluster, and Cys^565^, which is located in the cleft leading to the active site.

Not only aconitase but also other enzymes of the citric acid cycle are targeted by HNE. Zhao *et al.*, 2014 identified several HNE-modified mitochondrial proteins in mouse heart mitochondria after doxorubicin (DOX) treatment [174]. The majority of the identified proteins were related to mitochondrial energy metabolism. These included proteins in the citric acid cycle and electron transport chain.

###### 5.1.2.4.2. α-Enolase

The formation of HNE adducts with the multifunctional protein α-enolase was demonstrated by MS and confirmed by immunoblotting experiments, in HL-60 human leukaemic cells [175]. HNE caused a dose- and time-dependent reduction of the binding of plasminogen to α-enolase. As a consequence, HNE reduced adhesion of HL-60 cells to HUVECs (human umbilical vein endothelial cells) suggesting a potential role for HNE in the control of tumour growth and invasion.

Enolase is among the six enzymes which are excessively adducted by HNE in early AD (EAD) inferior parietal lobule (IPL) compared to age-related control brain [176]. These proteins play roles in antioxidant defense (manganese superoxide dismutase), neuronal communication and neurite outgrowth (dihydropyriminidase-related protein 2), and energy metabolism (α-enolase, malate dehydrogenase, triosephosphate isomerase, and F1 ATPase, α-subunit. These results are consistent with the hypothesis that LPO is an early event in the progression of AD.

##### 5.1.2.5. Isomerases

###### 5.1.2.5.1. Protein Disulfide Isomerase (PDI)

PDI is an abundant endoplasmic reticulum (ER)-resident chaperone and oxidoreductase that catalyzes formation and rearrangement (isomerization) of disulfide bonds, thereby participating in protein folding. PDI modification by HNE or oxidized LDL (oxLDL) inhibits its enzymatic activity and potentiates both ER stress (increased mRNA expression of the stress-regulated transcription factors CHOP (C/EBP homologous protein) and apoptosis by oxLDL [177].

###### 5.1.2.5.2. Peptidyl-Prolyl *Cis*/*Trans*-Isomerase A1 (Pin1)

Pin1, an enzyme that catalyzes the conversion of the peptide bond of pSer/pThr-Pro moieties in signaling proteins from *cis* to *trans*, is highly susceptible to HNE modification. This was demonstrated by Aluise *et al.*, 2013 upon incubation of purified Pin1 with HNE leading to Michael adducts at the active site residues His^157^ and Cys^113^ [178]. Additionally the authors confirmed Cys^113^ adducts in the Pin1 active site in MDA-MB-231 breast cancer cells treated with tagged HNE (8-alkynyl-HNE) and showed that knockdown of Pin1 in MDA-MB-231 cells partially protected the cells from HNE-induced toxicity.

##### 5.1.2.6. Ligases: Glutamine Synthetase

Glutamine synthetase is among the proteins modified by HNE in the retina as shown by Tanito *et al.*, 2006 [179]. By analyzing HNE-modified proteins increased after intense white light exposure nine proteins including voltage-dependent anion channel, enolase 1α, aldolase C, crystallins αA and βB3, heterogeneous nuclear ribonucleoprotein A2/B1, albumin, and glutamine synthetase were identified. The results indicate that HNE modifications of retinal proteins are specific to a particular set of proteins rather than random events on abundant proteins.

#### 5.1.3. Carriers

##### 5.1.3.1. Albumin

Toyokuni *et al.*, 2000 investigated the interaction of HNE with human serum albumin and found that it is rapidly quenched by human serum albumin (HSA) due to the covalent adduction to different accessible nucleophilic residues of the protein [180]. The molecular characterization of the covalent modifications led to the identification of eight Michael adducts and three Schiff bases involving nine nucleophilic sites. Cys^34^, His^146^, and Lys^199^ were found to be the most reactive HNE adduction sites. The latter adducted peptides were proposed as useful biomarkers of oxidative and carbonylation damage in humans [181]. In another MS investigation only those modified peptides were considered which were supported by high mass accuracy Orbitrap precursor ion measurements (high confidence hits) [182]. With HNE:HSA ratios of 1:1 and 10:1, 3 and 15 addition sites, respectively, were identified. This investigation confirmed previous work that Cys^34^, the only free cysteine, is the most reactive residue in HSA.

HNE-modified HSA is highly immunogenic eliciting high titre immunogen specific antibodies as was shown for systemic lupus erythematosus (SLE), a chronic autoimmune disease, which is primarily characterized by increased levels of autoantibodies, predominantly against *double stranded DNA* [183]. A preferential binding of SLE autoantibodies to HNE-modified HSA as compared to native HSA or native DNA was shown and indicates a potential role of HNE-modified HSA in SLE etiopathogenesis.

##### 5.1.3.2. Hemoglobin and Myoglobin

While hemoglobin (Hb) can be modified by HNE in cell-free experiments, HNE-Hb adducts were not detected in erythrocytes under oxidative stress induced by gamma-irradiation [184]. In myoglobin (Mb) HNE forms adducts with His residues and destabilizes Mb redox state, affecting meat colour [185]. LC-ESI-MS/MS of chicken Mb reacted with HNE identified covalent adduction of His^64^ and His^93^ at pH 7.4.

##### 5.1.3.3. Liver and Adipocyte Fatty Acid-Binding Protein (FABP)

FABPs have been characterized as facilitating the intracellular solubilization and transport of long-chain fatty acyl carboxylates via noncovalent interactions. Sites of HNE adduction on mouse liver recombinant FABP (L-FABP) were mapped on apo (Lys^57^ and Cys^69^) and holo (Lys^6^, Lys^31^, His^43^, Lys^46^, Lys^57^ and Cys^69^) L-FABP [186]. These modifications revealed minor conformational changes in global protein structure of apo and holo L-FABP by molecular modeling simulations, but apparent differences were observed within the internal binding pocket.

The FABP of adipocytes is the first protein for which the crystal structure of its HNE adduct was determined. Recent work has shown that the adipocyte FABP is covalently modified by HNE *in vivo* on Cys^117^. To evaluate HNE binding and modification, the crystal structures of adipocyte FABP covalently and noncovalently bound to HNE have been solved to 1.9 Å and 2.3 Å resolution, respectively [187]. While HNE in the noncovalently modified protein is coordinated similarly to a carboxylate of a fatty acid, the covalent form shows a novel coordination through a water molecule at the polar end of the lipid. Other defining features between the two structures with HNE and previously solved structures of the protein include a peptide flip between residues Ala^36^ and Lys^37^ and the rotation of the side chain of Phe^57^ into its closed conformation [187]*.*

##### 5.1.3.4. Apolipoprotein B-100 (ApoB)

The adduct formation of HNE and 4-oxo-nonenal (ONE) resp. with ApoB demonstrate that reactive aldehydes generated by LPO can differ in their biological effects, and that these differences can be mechanistically explained by the structures of the protein adducts formed [188]. In a study to understand the pathobiology of reactive lipid aldehydes the effects of HNE and ONE on the transport and secretion of very low-density lipoprotein was studied. Physiologically relevant concentrations of HNE and ONE rapidly disrupted cellular microtubules of HepG2 cells in a concentration-dependent manner. ONE reduced ApoB secretion while HNE did not significantly impair secretion. Both HNE and ONE formed adducts with ApoB protein, but HNE adducts were detectable as mono-adducts, while ONE adducts were present as protein-protein cross-links [188]*.* Interestingly another apolipoprotein, namely apolipoprotein A-I, the major HDL protein, is not modified by HNE *in vitro*, while MDA is adducted to lysine residues under the same conditions [189].

##### 5.1.3.5. β-Lactoglobulin

No clear function has been identified for β-lactoglobulin, although it binds to several hydrophobic molecules, suggesting its role as a carrier. The strong suggestion is that the molecule exists primarily as a food source. β-Lactoglobulin was used as a model protein to study adduct formation *in vitro* during peroxidation of linolenic acid (LA) in the presence of Fe(II), and ascorbate [190]. By mass spectrometry the authors identified adducts including HNE-His Michael adducts, ONE-Lys 4-ketoamide, ONE-Lys pyrrolinone, and a Cys/His-ONE-Lys pyrrole cross-link. However, reversibly formed adducts, such as the HNE-Lys Schiff base were not present at detectable levels.

#### 5.1.4. Transporters and Channels

##### 5.1.4.1. Glutamate Transport Protein

HNE can inhibit glutamate transport *in vitro* and *in vivo*. Numerous *in vitro* and cell culture experiments indicate that oxidative damage decreases astrocyte glutamate transport activity. The hypothesis that LPO products impair glutamate and glucose transport *in vivo* has been tested by Ou *et al.*, 2002 [191]. Their findings indicate that LPO products that irreversibly modify protein lysyl residues cause a two- to sixfold elevation in extracellular glutamate in striatum and cerebral cortex of rats undergoing microdialysis and that LPO product-evoked extracellular glutamate appeared to be derived from nonneuronal sources. These results support the hypothesis that oxidative damage leads to inhibition of glutamate transport and thereby contributes to the progression of neurodegenerative diseases. Mattson and Chan [192] showed that in AD Aβ can induce membrane LPO and the formation of HNE. Aβ thereby impairs the function of glutamate and glucose transporters, membrane ion-motive ATPases, and can enhance calcium influx through voltage-dependent and ligand-gated calcium channels. In sporadic amyotrophic lateral sclerosis (ALS) patients increased modification of proteins by HNE has been reported in the lumbar spinal cord compared to neurologically normal controls. Biochemical analysis revealed that one of the proteins modified by HNE was the astrocytic glutamate transporter EAAT2 [193]. The authors conclude that the function of proteins modified by HNE can be severely compromised leading to impairment of glutamate transport, and excitotoxic motor neuron degeneration in ALS.

For a homolog of HNE, 4-hydroxyhexenal, similarly the impairment of glutamate transport in astrocytes has been demonstrated [194].

##### 5.1.4.2. α-Synuclein (α-Syn)

α-Syn is a small protein that is abundant in various regions of the brain [195]. Although the physiological function of α-Syn is not well understood, its function is likely to involve presynaptic vesicle pool size and neurotransmitter release [196,197], and vesicle recycling [198]. One of the pathological hallmarks of Parkinson disease (PD) is the presence of intracellular inclusions called Lewy bodies that consist of aggregates of α-Syn (for review see [199]). Xiang *et al.*, 2013 [200] investigated posttranslational modifications (PTMs) of α-Syn caused by oxidative stress, including modification by HNE (HNE-α-Syn), nitration (N-α-Syn), and oxidation (O-α-Syn), which have been reported to promote oligomerization of α-Syn and found that modification of α-Syn by HNE increased dopaminergic toxicity by increasing the interaction of extracellular α-Syn with neurons. In another study [201], Bae *et al.*, 2013 investigated the oligomerization of recombinant human α-Syn via HNE adduct formation at the lysine and histidine residues and found that (a) HNE-induced α-Syn oligomers are distinct from amyloid fibrils at both conformation and ultrastructure levels; (b) the HNE-induced oligomers are capable of seeding the amyloidogenesis of monomeric α-Syn under *in vitro* conditions; (c) after treatment with HNE both the translocation of α-Syn into vesicles and the release of this protein from cells were increased. The HNE- or ONE-induced α-Syn oligomers have been characterized by Nasstrom *et al.*, 2011 [202]. Both oligomers are rich in beta-sheet structure and have a molecular weight of about 2000 kDa. Atomic force microscopy analysis revealed that neither oligomer type polymerized into amyloid-like fibrils despite prolonged incubation. According to findings of Shibata *et al.*, 2010 both ONE- and HNE-induced α-Syn oligomers were cytotoxic when added exogenously to a neuroblastoma cell line, but HNE-induced α-Syn oligomers were taken up by the cells to a significantly higher degree [203].

##### 5.1.4.3. Sarco/Endoplasmic Reticulum Ca^2+^-ATPase (SERCA1a)

HNE has a dual effect on Ca^2+^ transport through sarcoplasmic reticulum membranes from rabbit fast-twitch skeletal muscle as reported by Hortigon-Vinagre *et al.*, 2011 [204]. The authors showed that exposure of the membranes to HNE resulted in inhibition of the maximal ATPase activity and Ca^2+^ transport ability of SERCA1a, the Ca^2+^ pump in these membranes, reduced ATP binding and phosphoenzyme formation from ATP, whereas Ca^2+^ binding to the high-affinity sites was altered to a lower extent, and that HNE reacted with Lys^515^ within the nucleotide binding pocket of SERCA1a.

##### 5.1.4.4. Transient Receptor Potential Vanilloid 1 (TRPV1)

TRPV1 is a nociceptive, Ca^2+^-selective ion channel activated by capsaicin, heat, and protons. The function of TRPV1 is detection and regulation of body temperature. In addition, TRPV1 provides sensing of scalding heat and pain (nociception). It has been reported that activation of TRPV1 expressed in esophageal mucosa is involved in gastroesophageal reflux disease (GERD) or in nonerosive GERD symptoms [205]. Capsaicin has been shown to significantly increase the production of HNE-modified proteins in Het1A cells, and IL-8 production in capsaicin-stimulated Het1A cells was enhanced by synthetic HNE treatment. By analyzing protein modifications it turned out that TRPV1 was modified by HNE [206]. It was therefore concluded that TRPV1 functions in chemokine production in esophageal epithelial cells, and this function may be regulated by HNE via posttranslational modification of TRPV1*.*

##### 5.1.4.5. Dopamine Transporter

LoPachin *et al.*, 2009 characterized the synaptosomal toxicity of HNE and evaluated the role of putative nucleophilic amino acid targets and were able to show that HNE exposure of striatal synaptosomes inhibited (3)H-dopamine membrane transport and vesicular storage which was paralleled by decreases in synaptosomal sulfhydryl content [206]. Furthermore they demonstrated that the sulfhydryl thiolate state was the HNE target, that the rate of adduct formation was pH-dependent and that *N*-acetyl-l-cysteine, but not *N*-acetyl-l-lysine or β-alanyl-l-histidine, reduced *in vitro* HNE neurotoxicity.

#### 5.1.5. Receptors

##### 5.1.5.1. Platelet-Derived Growth Factor Receptor-β (PDGFR-β)

The PDGFR-β signaling pathway regulates smooth muscle cell (SMC) migration and proliferation and plays a role in the vascular wall response to injury. HNE and oxidized low-density lipoprotein (oxLDL) induce a dual effect on PDGFR-β signaling [207]. Short-term incubation of SMC with HNE or oxLDL induces PDGFR-β pathway activation via the formation of PDGFR-adducts and oxidative stress increase. In contrast, long-term incubation triggers a desensitization of PDGFR to its own agonist, leading to a progressive inhibition of PDGF-β-mediated signalling and proliferation, resulting from decreased PDGF binding and inhibition of PDGFR-β tyrosine kinase activity. PDGFR inhibition was associated with increased formation of HNE- and acrolein- PDGFR-adducts. PDGFR-β adducts were detected in aortae of apolipoprotein-deficient mice, in hyper-cholesterolemic rabbits and in human carotid plaques. The aldehyde scavengers dinitrophenylhydrazine and hydralazine prevented both HNE- and oxLDL-induced structural modification and PDGFR-β signaling dysfunction in cells and *in vivo* [207]. In conclusion, PDGFR-β acts as a sensor for both oxidative stress and oxidized lipids in atherosclerotic areas and its progressive inhibition by HNE or acrolein may contribute to defective SMC proliferation, and decrease the stability of a vulnerable plaque.

##### 5.1.5.2. Lectin-Like Oxidized Low-Density Lipoprotein Receptor-1 (LOX-1)

LOX-1 is an endothelial scavenger receptor that is important for the uptake of oxLDL. However, the precise structural motifs of oxLDL that are recognized by LOX-1 are unknown. By investigating the ability of BSA modified by lipid peroxidation to compete with AcLDL (acetylated low-density lipoprotein), Kumano-Kuramochi *et al.*, 2012 showed that HNE-modified proteins most potently inhibited the uptake of AcLD, that both the modification of BSA and the oxidation of LDL resulted in the formation of HNE-histidine Michael adducts, that the HNE-histidine adduct inhibited the uptake of AcLDL in a dose-dependent manner, and that the HNE-histidine adduct stimulated the formation of ROS and activated extracellular-signal-regulated kinase 1/2 (ERK 1/2) and NFκB thus initiating endothelial dysfunction and leading to atherosclerosis [208].

##### 5.1.5.3. Toll-Like Receptor 4 (TLR4)

Toll-like receptors (TLRs) detect invading microbial pathogens and initiate immune responses as part of host defense mechanisms. They also respond to host-derived substances released from injured cells and tissues to ensure wound healing and tissue homeostasis. Dysregulation of TLRs increases the risk of chronic inflammatory diseases and immune disorders. Inflammatory events are often accompanied by oxidative stress which generates lipid peroxidation products such as HNE. Therefore, Kim *et al.*, 2009 investigated whether HNE affects TLR activation and found that HNE blocked LPS (a TLR4 agonist)-induced activation of NFκB and IRF3 as well as expression of IFN-β, IP-10, RANTES and TNFα [209]. Furthermore, they were able to demonstrate that HNE suppressed both ligand-induced and ligand-independent receptor dimerization and that HNE formed adducts with cysteine residues of synthetic peptides derived from TLR4 suggesting that the reactivity of HNE with sulfhydryl moieties is implicated in the inhibition of TLR4 activation. Since inhibition of TLR4 activation by HNE also resulted in down-regulation of the phagocytic activity of macrophages it can further be concluded that HNE blocks TLR4-mediated macrophage activation and phagocytic functions.

#### 5.1.6. Cytoskeletal Proteins

##### 5.1.6.1. Tau Proteins

Tau proteins are microtubule-associated proteins found in neurons in the brain. These proteins interact with tubulin to stabilize microtubules and promote tubulin assembly into axonal microtubules. Tau has two ways of controlling microtubule stability: isoforms and phosphorylation. The formation of tau-containing neurofibrillary tangles is a major feature of AD and other neurodegenerative diseases. Fibers are correlated with disease severity and they have been implicated as playing a direct role in disease pathophysiology. Biochemical findings show that tau oxidative modifications are regulated by phosphorylation and that tau found in neurofibrillary tangles is oxidatively modified, suggesting that it is closely linked to the biology, not toxicity, of AD [210]. Several studies support a link between tau protein phosphorylation and adduction of tau by reactive carbonyls. The phosphorylation-dependent adduction of tau by carbonyl products resulting from LPO creates the neurofibrillary tangle-related antigen, Alz50. To determine whether epitopes of carbonyl-modified tau are major conformational changes associated with neurofibrillary tangle formation, seven distinct antibodies raised against neurofibrillary tangles have been examined that recognize unique epitopes of tau in AD. Consistently, all seven antibodies recognize tau more strongly (4- to 34-fold) after treatment of normal tau with HNE, but only when tau is in the phosphorylated state [210]. *In vitro* HNE as well as several quinones facilitated the phosphorylated tubulin binding of tau into fibrillar polymers which have a morphology very similar to that of paired helical filaments present in the brains of AD patients [211]. These findings not only support the idea that oxidative stress is involved in neurofibrillary tangle formation occurring in brains of AD patients, but also show that HNE modifications of tau promote and contribute to the generation of the major conformational properties defining neurofibrillary tangles [210].

##### 5.1.6.2. Ankyrin

Ankyrin is an adaptor protein in the membrane cytoskeleton of erythrocytes. The modification of ankyrin is pathologically relevant in malaria [212], however, the molecular basis for the prevalence of blood group 0 in regions where malaria is endemic remains unclear [212]. Mendez *et al.*, 2012 [212] therefore investigated whether there are differences in carbonylated membrane proteins between the different blood groups and found that group 0 blood showed a reduced protein oxidation pattern compared to groups A, B and AB. By examining HNE modified proteins, ankyrins were found to be differentially carbonylated in group 0 upon malaria infection as compared to A and B groups.

##### 5.1.6.3. Spectrins

Both and spectrin of the cytoskeleton of erythrocytes (RBC) are targets of HNE [213]. Spectrin strengthens the RBC membrane through its direct association with membrane lipids and through protein-protein interactions. Spectrin loss on the other hand reduces membrane stability and results in various types of hereditary spherocytosis. However, less is known about acquired spectrin damage. Arashiki *et al.*, 2010 were able to show that α- and β-spectrin in human RBC are the primary targets of HNE, *i.e.*, the level of HNE adducts in spectrin (particularly α-spectrin) was increased following HNE treatment of RBC membrane ghosts [213]. In contrast, ghost preparation in the presence of MgATP reduced HNE adduct formation, with preferential β-spectrin modification and increased cross-linking of the HNE-modified spectrins resulted in selective HNE-spectrin adduct formation. These findings were interpreted in terms of preferential HNE adduction in spectrin at the interface between the skeletal proteins and lipid bilayer. A combined protocol of immune-detection, peptide enrichment, mass spectrometry, and *de novo* protein sequencing was applied to study the adduction sites in human β-spectrin [214]. HNE-lysine and HNE-histidine Michael adducts were detected in β-spectrin under physiological conditions.

#### 5.1.7. Chaperones: Heat Shock Proteins 70 and 90

In a rat model of chronic alcohol-induced oxidative stress [91] Carbone *et al.*, 2004 investigated the modification of both cytosolic Hsp72, the inducible variant of Hsp70, and Hsp90 by HNE. By applying mass sprectrometrical analysis they were able to show that Hsp72 treated with 10 and 100 µM HNE caused adduct formation at Cys^267^ in the ATPase domain of the chaperone. Cys^572^ was found to be a site for HNE modification in Hsp90 [90]. HNE adducted Hsp70 has been identified in neuronal necrotic cell death [215] and in ALS an age-related, fatal motor neuron degenerative disease [216]. Although multiple mechanisms contribute to the pathogenesis of motor neuron injury in ALS, it is likely that oxidative stress plays a significant role in the amplification, and possibly the initiation, of the disease. HNE levels are increased in spinal cord motor neurons of ALS patients, indicating that LPO is associated with the motor neuron degeneration in ALS. Three significantly HNE-modified proteins were found in the spinal cord of G93A-SOD1 transgenic mice: besides Hsp70 dihydropyrimidinase-related protein 2, and possibly α-enolase.

HNE-modified Hsp70 is much more vulnerable to calpain cleavage [217]. The calpain-cathepsin cascade is mediating necrotic neuronal death from simpler organisms to primates. The main event of this cascade is calpain-mediated lysosomal rupture and the resultant release of lysosomal cathepsins into the cytoplasm. However, the *in vivo* substrate of calpain for inducing lysosomal destabilization still remains completely unknown. Recent data obtained with post-ischemic hippocampal CA1 tissues and glaucoma-suffered retina from primates suggests that Hsp70.1 might be the *in vivo* substrate of activated µ-calpain at the lysosomal membrane of neurons. Hsp70.1 is known to stabilize lysosomal membrane by recycling damaged proteins and protect cells from oxidative stresses [217]. Sahara *et al.*, 2010 studied the molecular interaction between activated µ-calpain and the lysosomal Hsp70.1 in monkey hippocampal CA1 neurons after ischemia-reperfusion insult and found that Hsp70.1 in CA1 tissue is an *in vivo* substrate of activated µ-calpain and that Hsp70.1 carbonylated by HNE or hydrogen peroxide is much more vulnerable to calpain cleavage [217]. These data further suggest that Hsp70.1 can become a target of the carbonylation by HNE, and Hsp70.1 is a modulator of calpain-mediated lysosomal rupture/permeabilization after ischemia-reperfusion injury.

The adduction of Hsp90 by HNE has been studied by protein-selective capture [218] in RKO colorectal cancer cells and identified His^450^ and His^490^ adducts of Hsp90α. Five other histidine residues were also adducted on Hsp90β: His^171^, His^442^, His^458^, His^625^ and His^632^.

Both Hsp70 and Hsp90 are together with PDI, liver FABP and the protein-serine/threonine kinases ERK1 and ERK2 targets of HNE in early stages of ALD [219]. The adduction leads to inhibition of these enzymes and it was concluded that inhibition of Hsp70, Hsp90 and PDI function could be involved in initiation of the early phases of ER stress contributing to stimulation and accumulation of hepatic lipids. Likewise, impairment of L-FABP activity could also disrupt lipid transport contributing to steatosis. The modification and inhibition of Erk1/2 by HNE may in addition contribute to the decreased hepatocellular proliferation associated with ALD [219].

The electrophilic adduction of Hsp70 and Hsp90 by HNE appears to mediate in part the activation of heat shock factor 1 (HSF1), which is normally maintained in an inactive cytosolic complex [220]. This is one of the mechanisms, by which cellular responses to HNE are elicited, as was revealed by a systems analysis approach of protein modification and cellular responses induced by electrophile stress aiming to define the chemistry of protein modification and its biological consequences using lipid-derived α,β-unsaturated aldehydes such as HNE as model electrophiles. In this global approach, two large data sets were analyzed: one represented the identity of proteins modified over a wide range of electrophile concentrations, and the second comprised changes in gene expression observed under similar conditions. Informatics tools showed theoretical connections based primarily on transcription factors hypothetically shared between the two data sets, downstream of adducted proteins and upstream of affected genes [220]. This technique identified a multitude of HNE targets such as several transcription factors as potential mediators of the cellular response to HNE-adducted proteins. Among these, HSF1 was confirmed as a sensitive and robust effector of HNE-induced changes in gene expression. Among the genes induced by HSF1, Bcl-2- associated athanogene 3 (BAG3) is notable for its actions in promoting cell survival through stabilization of antiapoptotic Bcl-2 proteins, thus having a critical role in mediating cellular protection against electrophile-induced death [220].

The ER homolog of HSP70 is glucose-regulated protein 78 (GRP78). It plays a critical role in the cellular response to ER stress by serving as a chaperone assisting protein folding and by regulating the signaling of the unfolded protein response (UPR). GRP78 revealed a marked propensity for Lys and His adduction by HNE within the ATPase domain and a relative paucity of adduct formation within the peptide-binding domain [221]. Consistent with these findings, a concomitant dose-dependent decrease in ATP-binding and ATPase activity was observed without any discernible impairment of chaperone function.

#### 5.1.8. Uncoupling Proteins 2 and 3 (UCP2 and UCP3)

Most studies with HNE were focused on mitochondrial UCP2 and UCP3. Mitochondria are potent producers of cellular superoxide from complexes I and III of the electron transport chain, and mitochondrial superoxide production is a major cause of the cellular oxidative damage that may underlie degradative diseases and aging. This superoxide production is very sensitive to the proton motive force, so it can be strongly decreased by mild uncoupling of oxidative phosphorylation. Superoxide and the downstream LPO products including hydroxyalkenals such as HNE, are potent activators of proton conductance by mitochondrial uncoupling proteins such as UCP2 and UCP3, although the mechanism of activation has yet to be established. It was therefore suggested that superoxide releases iron from aconitase, leading to LPO and the release of molecules such as HNE that covalently modify and activate the proton conductance of UCPs and other proteins such as the adenine nucleotide translocase [222]. UCP2 and UCP3 do not catalyse proton leak in the absence of such acute activation. They can also catalyse export of fatty acid and other anions, although the relationship of anion transport to proton transport remains controversial [223]. The notion that HNE plays a role in the function of UCPs to minimize superoxide formation is supported by a study on plant mitochondria. HNE and a structurally related compound, trans-retinal, stimulated proton conductance in potato mitochondria, that is inhibitable by GTP, a characteristic of UCP. Proof that the effect of HNE and trans-retinal are mediated by UCPs was provided by examination of proton conductance in transgenic plants overexpressing UCP. An increase in UCP content resulted in a modest but significant decrease in the rate of superoxide production [224]. A hypothesis has been forwarded for the main function of uncoupling proteins: to cause mild uncoupling to diminish mitochondrial superoxide production, hence protecting against disease and oxidative damage at the expense of a small loss of energy. This simple feedback loop would constitute a self-limiting cycle to protect against excessive superoxide production. A more recently evolved role of UCP2 is perhaps as part of a signaling pathway to regulate insulin secretion in pancreatic beta cells [222,225,226].

Recently it was also shown that fatty acids are key players in HNE-mediated activation of UCP1 and UCP2 [227]. The molecular mechanisms of HNE action were investigated by evaluating the separate contributions of lipid and protein phases of the membrane and by comparing UCP1 and UCP2, which were reconstituted in planar lipid bilayers. Via this approach it was demonstrated that HNE does not directly activate either UCP1 or UCP2. Instead, it strongly potentiates the membrane conductance increase (G(m)) mediated by different long-chain fatty acids in UCP-containing and in UCP-free membranes. This G(m) increase is concentration-dependent and exhibits a typical saturation kinetics. In addition, for the amoeba *Acanthamoeba castellani* it was shown that the HNE induced UCP-mediated mitochondrial uncoupling is GTP-sensitive [228].

HNE is able to increase the proton conductance of the inner mitochondrial membrane through effects not only on uncoupling proteins but also the adenine nucleotide translocase (ANT). To clarify the relative contribution of the two carriers to these effects mitochondria were isolated from skeletal muscle and heart of wild-type and Ucp3 knockout (Ucp3KO) mice [229]. To increase UCP3 expression, some mice were i.p. injected with LPS which did not change basal proton conductance. HNE increased the proton conductance of skeletal muscle and heart mitochondria. In skeletal muscle, this increase was lower in Ucp3KO mice and higher in LPS-treated wild-type mice, and was partially abolished by GDP (UCPs inhibitor) and completely abolished by carboxyatractylate (ANT inhibitor) or addition of both inhibitors. GDP had no effect on HNE-induced conductance in heart mitochondria, but carboxyatractylate or administration of both inhibitors had a partial effect. GDP-mediated inhibition of HNE-activated proton conductance in skeletal muscle mitochondria was not observed in Ucp3KO mice, indicating that GDP is specific for UCP3, at least in muscle. It was therefore concluded that, in skeletal muscle, HNE-induced increase in proton conductance is mediated by UCP3 (30%) and ANT, whereas in the heart the increase is mediated by ANT and other carriers, possibly including UCP3 [229].

#### 5.1.9. Growth Factors: Platelet-Derived Growth Factor (PDGF)

Jin *et al.*, 2013 investigated breast cancer-associated changes of HNE, GSH, nitrotyrosine and halotyrosine adducts in 27 secreted proteins, for a total of 108 candidate biomarkers in plasma samples for both cases and benign controls and identified HNE-modified PDGF and GSH-modified ceruloplasmin which were significantly altered in samples from cancer patients [230]. The authors concluded that these changes may reflect redox changes in breast cancer, and that HNE-modified PDGF may be useful as plasma biomarker in breast cancer.

#### 5.1.10. Peptide Hormones

##### 5.1.10.1. Insulin

Human insulin is a target of both HNE and 4-hydroxy-hexenal (HHE) [231]. This was demonstrated by incubation of insulin in the presence of HNE or HHE and adduct analysis. The formation of covalent adducts on insulin was analyzed by mass spectrometry analysis and revealed one to five Michael adducts on insulin. Furthermore the authors report that glucose uptake in 3T3-L1 and L6C5 cells was significantly reduced after treatment with adducted insulin compared to native insulin, indicating that the formation of HNE- and HHE-Michael adducts significantly disrupted the biological activity of insulin.

##### 5.1.10.2. Angiotensin II

Another peptide hormone adducted by HNE is angiotensin II [232]. The octapeptide angiotensin II is the primary active hormone of the renin/angiotensin system (RAS) and has been implicated in various cardiovascular diseases. Numerous structure activity relationship studies have identified Asp^1^, Arg^2^, and His^6^ of Ang II to be critical for its biological activity and receptor binding [232]. Subsequently, it has been elaborated that the oxidative modifications on the *N*-terminus of Ang II disrupt interactions with Ang II type 1 receptor and aminopeptidase A (which cleaves the N-terminal Asp residue of Ang II to generate Ang III), which could affect the regulation of cardiovascular function [232].

#### 5.1.11. Extracellular Matrix Proteins: Collagen

HNE forms adducts with type II collagen (Col II). El-Bikai *et al.*, 2010 addressed the question whether interactions between human osteoarthritic chondrocytes and HNE-modified Col II affect cell phenotype and functions and to determine the protective role of carnosine (CAR) treatment in preventing these effects [233]. Their major findings were that Col II modification by HNE at a molar ratio (MR) approximately 1:20, strongly induced ICAM-1, α1β1-Integrin and metallo-proteinase-13 (MMP-13) expression as well as extracellular signal-regulated kinases 1 and 2 (ERK1/2) and NFκB-p65 phosphorylation without having an impact on cell adhesion and viability or Col II expression. In contrast, Col II modification with HNE at MR approximately 1:200, altered chondrocyte adhesion by evoking cell death and caspase-3 activity, and the inhibition of α1β1-integrin and Col II expression as well as ERK1/2 and NFκB-p65 phosphorylation. At the same time a release of PGE_2_, expression of COX-2 and p38 MAPK phosphorylation were observed. All these effects were prevented by CAR, an HNE-trapping drug (see chapter 5.1.1.2., p.20). The authors concluded that HNE-binding to Col II results in multiple abnormalities of chondrocyte phenotype and function, suggesting its contribution in osteoarthritis development [233].

#### 5.1.12. Histones: Histone-H2A

Histones are DNA associated nucleoproteins, which adopt different structures under oxidative stress. A study was undertaken to test the role of HNE-modified histone-H2A (HNE-H2A) in *systemic lupus erythematosus* (SLE) [234]. The data revealed that HNE-mediated LPO in histone-H2A caused alteration in histidine, lysine and cysteine residues. In addition, protein carbonyl contents were also high in HNE-H2A. The specificity of autoantibodies from SLE patients were analyzed towards HNE-H2A and their results were compared with sex- and age-matched controls. SLE autoantibodies showed preferential binding to HNE-H2A in comparison with histone-H2A*.* In addition, HNE-H2A was also detected in SLE peripheral blood mononuclear cells suggesting a likely role of HNE-H2A in the initiation/progression of SLE [234]*.*

### 5.2. Reactions with Lipids

The phospholipids phosphatidyl-ethanolamine (PE) and—to a lesser extent—phosphatidyl-serine, which contain a primary amino group, can undergo both Michael addition and Schiff-base formation with HNE [235]. The Schiff-base of PE partly undergoes cyclization to a pyrrole derivative, and PEs modified by HNE and other aldehydes induce monocyte adhesion to cultured endothelial cells.

LPO generates a large family of aldehyde-modified PEs (al-PEs), many of which have the potential to drive inflammation [236], *i.e.*, gamma-ketoaldehydes (isolevuglandins, IsoLGs) form inflammatory mediators by modifying the ethanolamine headgroup of PEs. When the ability of al-PEs was tested to induce THP-1 monocyte adhesion to cultured endothelial cells, it was found that PEs modified by HNE, ONE and MDA induced adhesion with potencies similar to those of PEs modified by IsoLGs, while PEs modified by acrolein or by glucose were only partial agonists for adhesion [236].

### 5.3. Reactions with Cofactors and Vitamins

#### 5.3.1. Vitamin C (Ascorbic Acid)

Of interest is the observation that HNE, like other LPO-derived electrophiles, can undergo ascorbylation. Vitamin C can form a Michael-type conjugate with HNE *in vitro* [237,238]. The reaction has been described as a Michael addition of an activated C-H group of ascorbic acid to the C=C double bond of HNE followed by cyclisation to produce a hemiacetal [48]. Ascorbic acid is able to promote the detoxification and elimination of HNE in human monocytic THP-1 leukemia cells [239]. In this process adduct formation of ascorbic acid with HNE is only a minor pathway. It was therefore suggested that the protective effect of ascorbate against HNE cytotoxicity is largely via modulation of multidrug resistant protein (MRP)-mediated transport of GSH-HNE conjugate metabolites.

#### 5.3.2. Pyridoxamine

Also the cofactor pyridoxamine is an HNE target. In search for new scavengers of carbonyl compounds, the amino group of pyridoxamine was demonstrated to react faster with HNE than the amino group of *N*-acetyl lysine, but much slower than a series of 4-ketoaldehydes with oxopentanal as model compound [240].

#### 5.3.3. Lipoic Acid

The reduced form of lipoic acid contains two thiol groups which can be adducted by HNE. Both lipoic acid and its redox enzyme lipoamide dehydrogenase (LADH) are targets of HNE in AD brain. The AD brain shows increased levels of LPO products, including HNE. The levels of the HNE-modified lipoic acid in brain of subjects with AD and age-matched controls were therefore measured by Hardas *et al.*, 2013 [241]. Lipoic acid is a key co-factor for a number of proteins including pyruvate dehydrogenase and α-ketoglutarate dehydrogenase. Decreased levels of HNE-lipoic acid in the AD brain were observed in this study compared to that of age-matched controls. It was further demonstrated that both LADH levels and activities were significantly reduced in AD brain compared to age-matched control. The authors conclude that these data are consistent with a two-hit hypothesis of AD: oxidative stress leads to LPO that, in turn, causes oxidative dysfunction of key energy-related complexes in mitochondria, triggering neurodegeneration, and they suggest that lipoic acid supplementation could be a potential treatment for the observed loss of cellular energetics in AD to potentiate the antioxidant defense system to prevent or delay oxidative stress in and progression of this disorder [241].

### 5.4. Reactions with Nucleic Acids

The current knowledge on DNA damage induced by endogenously produced reactive aldehydes such as HNE, MDA, acrolein, crotonaldehyde and methylglyoxal in relation to the pathophysiology of human diseases has been reviewed by Voulgaridou *et al.*, 2011 [242]. Minko *et al.*, 2009 [243] gave an overview of the chemistry and biology of DNA containing 1,N^2^-deoxyguanosine adducts of the α,β-unsaturated aldehydes HNE, acrolein, and crotonaldehyde.

HNE is genotoxic, e.g., for hepatocytes [244] and cerebral endothelial cells [245]. HNE in a concentration of 0.1 µM causes elevated levels of sister chromatid exchanges in primary hepatocytes, 1 µM causes elevated levels of chromosomal aberrations and at 10 µM the formation of micronuclei is observed. HNE is thought to contribute to the low level of DNA adducts which are abundant both in untreated rodent and human genomes. Two pathways are considered to be responsible for the mutagenicity of HNE: one route is the formation of an adduct by direct interaction with the guanosine moiety of DNA (Figure 4). The reaction of HNE with DNA gives four diastereomeric 1,N^2^-γ-hydroxypropano adducts of deoxyguanosine; background levels of these adducts have been detected in animal tissue. Stereospecific synthesis of these four adducts at the nucleoside level have been accomplished. In addition, a versatile strategy for their site-specific incorporation into oligonucleotides has been developed [246].

Reaction of HNE with calf thymus DNA resulted in a pair of diastereomeric adducts. Background levels of the 1,N^2^-propane adduct of guanine (HNE-dG) in rat tissues are in the range of 18–158 adducts per 10^9^ nucleotides with relatively high levels in the liver [247]. The guanine adducts can form interstrand cross-links. When placed opposite dC in the 5'-CpG-3' sequence, the (6S,8R,11S) diastereomer of the HNE-dG forms a N^2^-dG:N^2^-dG interstrand cross-link [246,248]. Its structure was refined by Huang *et al.*, 2010 and 2011 showing that the crosslink is a N^2^-dG:N^2^-dG carbinolamine [249,250]. These cross-linked -enal adducts are likely to contribute to the genotoxic effects of both HNE and acrolein. These cross-linked adducts occur at levels that are similar to 1%–2% of the levels of the monomeric 1,N^2^-dG adducts in calf thymus DNA treated with either -enal.

Besides HNE also acrolein can form a cyclic adduct of deoxyguanosine (Acr-dG) in DNA. *In vivo* levels of Acr-dG in DNA are at least two orders of magnitude higher than those of the HNE adduct HNE-dG [251,252]. In addition to the facile reaction with acrolein, the higher levels of the acrolein adduct *in vivo* are due to a lower rate of repair.

The consequences of cellular formation of LPO products, particularly HNE, in terms of genomic stability have been reviewed by Winczura *et al.*, 2012 [253]. Elimination from DNA of LPO-induced lesions is executed by several repair systems: base excision repair (BER), direct reversal by AlkB family proteins, nucleotide excision repair (NER) and recombination. NER and recombination are involved in repair of HNE adducts to DNA bases in Escherichia coli [254]. According to the authors Acr-dG in a plasmid DNA is repaired by NER proteins, but it is repaired at a much slower rate than HNE-dG in human colon cell extracts, and the slow repair of Acr-dG is likely due to poor recognition/excision of the lesions in DNA. Using a plasmid DNA containing both adducts it was shown that the repair of Acr-dG is significantly inhibited by HNE-dG. In contrast, the repair of HNE-dG is not much affected by Acr-dG [251].

Interestingly, the 1,*N*-2-propane adduct of guanine in DNA can be bypassed during replication by Sulfolobus solfataricus P2 DNA polymerase IV [255].

The role of damage-specific DNA polymerases in mutagenesis induced by HNE was studied in M13mp18 phage by testing survival and mutation spectra in the lacZα gene [256]. The phage was grown in uvrA(−) Escherichia coli strains, carrying one, two or all three SOS DNA polymerases. When Pol IV was the only DNA SOS polymerase in the bacterial host, survival of HNE-treated M13 DNA was similar to, but mutation frequency was lower than in the strain containing all SOS DNA polymerases. When only Pol II or Pol V were present in host bacteria, phage survival decreased dramatically. Simultaneously, mutation frequency was substantially increased, but exclusively in the strain carrying only Pol V suggesting that induction of mutations by HNE is mainly dependent on Pol V [256]*.*

The second route for the mutagenicity of HNE putatively involves the oxidation of HNE to its epoxide. The resulting etheno adducts have been found in human lung tissue [257]. The reaction mechanism was studied by Lee *et al.*, 2005 [258]. Analysis of the reaction between 4-hydroperoxy-2-nonenal (HPNE) and 2'-deoxyguanosine (dGuo) revealed the formation of 1,N^2^-etheno-dGuo as well as heptanone-etheno-dGuo and trace amounts of dihydroxyheptane-etheno-dGuo. On the basis of these findings, it was concluded that HPNE, a primary product of LPO, is a major precursor to the formation of 1,N^2^-etheno-dGuo, and it was proposed that 1,N^2^-etheno-dGuo arises from the reaction of dGuo and HPNE via the intermediate formation of a cyclic hydroxy-ethano-epoxide derivative. In support of this assumption cytochrome P4502E1 appears to be responsible for the formation of HNE-etheno-DNA adducts in alcoholic liver disease [259].

Miscoding etheno-modified DNA adducts including 1,N(6)-etheno-2'-deoxyadenosine (ε-dA) are excreted in urine, following elimination from tissue DNA. An ultrasensitive and specific immunoprecipitation/HPLC-fluorescence detection method was developed for quantifying ε-dA excreted in urine [260]. Levels in urine of Thai and European liver disease-free subjects were found to be in the range of 3–6 fmol ε-dA/µM creatinine. Subjects with inflammatory cancer-prone liver diseases caused by viral infection or alcohol abuse excreted massively increased and highly variable ε-dA-levels. Based on this pilot study it was concluded that (i) high urinary ε-dA-levels, reflecting massive LPO-derived DNA damage *in vivo* may contribute to the development of hepatocellular carcinoma (HCC); (ii) ε-dA-measurements in urine and target tissues should thus be further explored as a putative risk marker to follow malignant progression of inflammatory liver diseases in affected patients and that (iii) etheno adducts may serve as biomarkers to assess the efficacy of (chemo-) preventive and therapeutic interventions [260].

## 6. Formation of HNE in Mammalian Cells and Tissues

High HNE levels or increases of HNE are generally an indication for accelerated LPO. However, both the high reactivity of HNE and the rapid metabolism of HNE will overlap the formation rate of HNE, *i.e.*, even if the rates of LPO and of HNE formation are very high, the topical increasing HNE level does not fully reflect the increase of LPO and HNE formation rates due to the overlapping of formation and degradation. The dynamics of HNE turnover, which is not easy to analyze—e.g., with tracer kinetic measurements—is much higher than the simple increase rate of HNE concentration. Thus, when significant increases of HNE levels are found in organelles, cells, tissues, organs, or in whole organisms, one may assume that the total increase in LPO rate and in HNE formation rate is extremely high.

### 6.1. HNE Formation in Cellular and Organ Systems

The oxidative stress during posthypoxic reoxygenation of cell suspensions or postischemic reperfusion of organs was demonstrated by an increase of HNE levels—often paralleled by an enhancement of MDA or thiobarbituric acid-reactive substances (TBA-RS), decrease of GSH and increase of disulfides, too. Those changes were found in hepatocytes [261], renal tubular cells [262], heart [263,264,265], and small intestine [266]. In earlier studies, HNE increases were observed in hepatocyte suspensions under oxidative stress by CCl_4_ or ADP-iron [267]. An age-dependent increase of HNE in rats with traumatic brain injury was found in ipsilateral hippocampus one and seven days post injury [268]. Furthermore an increase of HNE was also found in hippocampus/parahippocampal gyrus, superior and middle temporal gyrus and cerebellum in mild cognitive impairment compared to age-matched control subjects [269]. This demonstrates that LPO occurs early in the pathogenesis of Alzheimer’s disease. Similar changes in Alzheimer’s disease and other neurodegenerative diseases were described by Negre-Salvayre *et al.*, 2010 [270].

**Figure 5:**
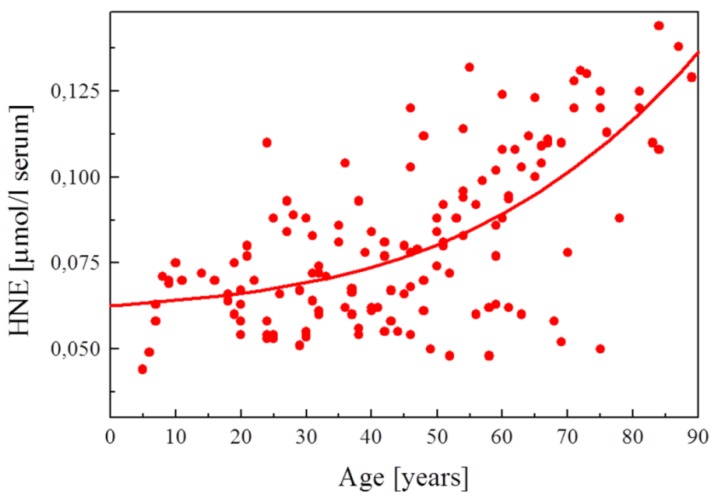
HNE plasma concentration in dependence on age of the blood donor (5 to 90 years).

Table 1 presents a list of cellular, tissue or organ concentrations of HNE in humans and other species. Additionally, Figure 5 demonstrates human blood plasma concentrations of HNE in dependence on age of the blood donors.

HNE formation was also measured in perfused organs. Changes of perfusion conditions lead to changes in HNE formation rate. The tissue concentration of HNE only partly represents the real HNE formation rate. The dynamics of HNE formation rate was of particular interest in the postischemic reperfusion syndrome. The real flux rate of intracellular HNE formation during normoxic perfusion, during ischemia and during postischemic reperfusion was estimated on the basis of tissue HNE concentration, HNE utilization rate at defined HNE tissue concentration and HNE washout by perfusate circulation in rat small intestine [266]. Such estimations led to intestinal HNE formation rates of about 20 nmol/g w.w./min at normoxia, 20 to 40 nmol/g w.w./min at ischemia, 100 nmol/g w.w./min at 10 min of postischemic reperfusion, and 20 nmol/g w.w./min at 60 min of reperfusion). These data showed that the highest increase of HNE formation occured during the early reperfusion phase following ischemia.

A significant increase of HNE concentration was found in human renal tubular cells [262] and in rat hepatocytes [261,271] during postanoxic reoxygenation in comparison with preanoxic control values. In parallel to the HNE increase decreases of energy rich nucleotides, in particular of ATP, of GSH and cell viability as well as electron-microscopic morphological alterations of cells were observed. In human monocytes, HNE increased after phagocytosis of the malarial pigment hemozoin reaching 4 pmol/10^6^ cells at 2 h (approximate intracellular concentration (AIE) 8 μM), 23 pmol/10^6^ cells at 5 h (AIE 46 μM), and 7.9 pmol/10^6^ cells (AIE 16 μM) at 12. A moderate increase in HNE, approximately 2 pmol/10^6^ cells (AIE 4 μM), was also observed after phagocytosis of anti-D IgG-opsonized erythrocytes. HNE in unfed controls was approximately 0.5 pmol/10^6^ cells (AIE 1 μM) [272]. The values which were measured in monocytes fed with hemozoin up to 50 µM were the highest intracellular concentrations which were ever measured. In some additional studies formation and metabolism of both HNE as well as MDA were compared [14].

**Table 1:** HNE concentrations in cells, tissues and organs.

Cells, Tissue	HNE-Concentration	Comment	Reference
Kidney tubular cells (human)	102 pmol/10^6^ cells	Praeanoxic control	[262]
	307 pmol/10^6^ cells	30 min reoxygenation after 60 min anoxia	
Hepatocytes (rat)	13 ± 5 pmol/10^6^ cells	Freshly prepared	[267]
	27 ± 18 pmol/10^6^ cells	CCl_4_-treated for 1 h	
	40 ± 14 pmol/10^6^ cells	ADP-Fe^3+^-treated for 1 h	
	0.43 pmol/5.2 × 10^6^ cells	Freshly prepared	[271]
	1.42 pmol/5.2 × 10^6^ cells	15 min reoxygenation after 60 min anoxia	
Monocytes (human)	0.5 pmol/10^6^ cells (1 μM) *	Basic value	[272]
	4 pmol/10^6^ cells (8 μM) *	Hemozoin fed at 2 h	
	23 pmol/10^6^ cells (46 μM) *	Hemozoin fed at 5 h	
	7.9 pmol/10^6^ cells (16 μM) *	Hemozoin fed at 12 h	
Small intestine (rat)	0.68 nmol/g w.w.	perfusion, normoxia	[266]
	3.02 nmol/g w.w.	reperfusion after 60 min ischemia	
Blood plasma (human)	0.074 ± 0.028 μM	194 healthy woman and men	[273]
	0.069 ± 0.015 μM	18 to 29 years aged	
	0.070 ± 0.014 μM	30 to 39 years aged	
	0.072 ± 0.020 μM	40 to 49 years aged	
	0.083 ± 0.020 μM	50 to 59 years aged	
	0.096 ± 0.022 μM	60 to 69 years aged	
	0.107 ± 0.027 μM	70 to 84 years aged	
	106.3 ± 65.8 ng/mL	*O*-pentafluorobenzyl oxime	[274]
Umbilical cord plasma (human)	0.3 μM	Full-term healthy neonates	[275]
	0.5 μM	Term neonates with acidosis	
	0.6 µM	Term neonates with asphyxia	
	0.1 µM	Preterm neonates (healthy)	
	0.4 µM	Preterm neonates (asphyxia)	

* The approximate intracellular HNE concentration is given in parentheses.

### 6.2. HNE in the Whole Healthy Organism

Increased HNE levels in blood serum and tissues were measured in various diseases in children and adults such as reperfusion syndrome, inflammatory diseases, rheumatological diseases (e.g., rheumatoid arthritis), lymphedema, AIDS, diabetes, and cystic fibrosis. Increased serum levels of HNE, MDA or other LPO products have also been demonstrated in several systemic autoimmune diseases in adults such as rheumatoid arthritis and systemic lupus erythematosus (SLE). Children suffering from SLE have significantly higher HNE levels (146 ± 14 nmol/L) compared to controls (61 ± 10 nmol/L) while children with the focal type of scleroderma (SCL) had HNE levels similar to the control group [276].

In general, basic HNE concentrations of blood serum (for adults and children except neonates) were found to be in the range of 0.05 and 0.15 μM [273]. These values were measured by means of the DTNB method and were in very good agreement with the plasma HNE concentration of 106.3 ± 65.8 ng/mL measured by means of another assay, the *O*-pentafluorobenzyl oxime method [274]. In about 200 healthy humans of ages ranging from 18 to 84 years plasma HNE was measured together with various oxidative stress parameters, giving a mean value of 0.074 μM [273]. Accelerated oxidation during ageing was demonstrated by increases of HNE, MDA, GSSG and by the slight decrease of erythrocytic GSH with age. In the group aged up to 30 years the mean HNE was 68.9 ± 15.0, whereas in the group aged more than 70 years the corresponding value was 107.4 ± 27.3 nmol/L [273]. HNE levels were measured in umbilical arterial cord blood samples from healthy, acidotic, and asphyctic neonates with a gestational age ranging from 26 to 41 weeks [275]. HNE in umbilical cord plasma of full-term healthy neonates amounted to about 0.3 μM. After perinatal complications, HNE increased significantly in the group of term newborns with acidosis to about 0.5 μM and in term neonates suffering from asphyxia to about 0.6 μM (*p* < 0.001 in comparison with the control group of healthy term newborns). In healthy preterm neonates with a gestational age from 34 to 36 weeks the mean HNE level was 0.1 μM. HNE increased up to 0.4 μM in preterm newborns after perinatal asphyxia (*p* < 0.001) [275].

The presence of protein-bound HNE *in vivo* has been assessed in various human tissues, including the human aorta with atherosclerotic lesions [277], nigral neurons in Parkinson disease [278], renal cell carcinomas [279], amyloid deposits in systemic amyloidosis [280], in brain from subjects with mild cognitive impairment [281], and trophoblast cells of preeclamptic placenta [282]. Furthermore, the demonstration of HNE protein adducts in human lung cells after ozone exposure are consistent with a potential role for HNE in the toxic effects of ozone in these cells [283].

Moreover, endogenous cyclic DNA adducts derived from HNE were associated with neurodegenerative diseases such as Alzheimer’s disease. A sensitive and selective capillary liquid chromatography nanoelectrospray isotope dilution mass spectrometric method was developed to identify and quantify endogenous cyclic DNA adducts derived from HNE with 2'-deoxyguanosine (HNE-dG) in human brain tissues [284].

### 6.3. Influence of Nutrition

Parallel to endogenous formation of HNE it can also be consumed with the diet. To assess human exposure to 4-hydroxy-2-alkenals in the diet, these lipid peroxidation products were monitored in vegetable oils, fish and shellfish [285] and the Korean daily exposure to 4-hydroxy-2-alkenals, excluding consumption from fried food, was calculated to be 4.3 μg/day (2.7 μg HNE and 1.6 μg HHE). Additionally, 4-hydroxy-2-alkenal intake from fried foods was calculated and amounted to more than 11.8 μg/day. Thus the combined exposure would be therefore, 16.1 μg/day corresponding to 0.3 μg/kg body weight/day for a 60 kg Korean adult [285]. It was also found that nutrition and various food constituents may have influence not only on HNE formation or intake, but also on HNE metabolism. Of course, this involves the sulfhydryl content of the food such as S-allyl-l-cysteine of garlic [286], but also other components. Dietary fibers are fermented by the gut flora to yield short chain fatty acids (SCFAs), which inhibit the growth of tumor cells, induce glutathione S-transferases, and protect cells from the genotoxic activity of HNE [287].

Beneficial effects of the probiotic VSL#3 (a medical food that delivers a high concentration of beneficial live bacteria) on parameters of liver dysfunction in chronic liver diseases were evaluated by Loguercio *et al.*, 2005 [288]. These effects were studied in non-alcoholic fatty liver disease, alcoholic liver cirrhosis, HCV-positive patients with chronic hepatitis without or with liver cirrhosis. Treatment with VSL#3 improved levels of HNE and MDA except in HCV-positive patients. Furthermore, routine liver damage tests and plasma S-NO levels were improved in all groups of patients. It was concluded that manipulation of the intestinal flora should be taken into consideration as possible adjunctive therapy in some types of chronic liver disease [288].

It was further shown that mean levels of HNE and MDA in rat brain tissues were decreased in rats fed soy-protein as a dietary antioxidant [289].

Data on the effect of green tea on LPO products formation and parameters of the antioxidative system of liver, blood serum and central nervous tissue of healthy young rats drinking green tea for five weeks were collected by Skrzydlewska *et al.*, 2002 [290]. The bioactive ingredients of green tea extract caused an increase in the activity of glutathione peroxidase and glutathione reductase and in the content of reduced glutathione as well as a marked decrease in lipid hydroperoxides, HNE and MDA in the liver while minor changes of the measured parameters were observed in the blood serum, but LPO products, particularly MDA, were significantly reduced. In the central nervous tissue the activity of superoxide dismutase and glutathione was found to be peroxidase while the activity of glutathione reductase and catalase were increased, and the level of lipid hydroperoxides, HNE and MDA significantly was found to be decreased after drinking green tea.

Beside of food uptake the uptake of environmental toxins may increase HNE concentrations in human tissues. Among the several converging factors leading to Parkinson disease, epidemiological studies indicate a correlation between Parkinson disease with living in a rural area and/or exposure to agricultural pesticides. In this context Leiphon and Picklo, 2007 demonstrated that mitochondrial aldehyde dehydrogenases are sensitive targets of pesticide inactivation and that pesticides such as maneb and benomyl can decrease the detoxification of HNE to HNA and therefore contribute to an increase of HNE in rat brain mitochondria [291].

## 7. Metabolism of HNE

**Figure 6:**
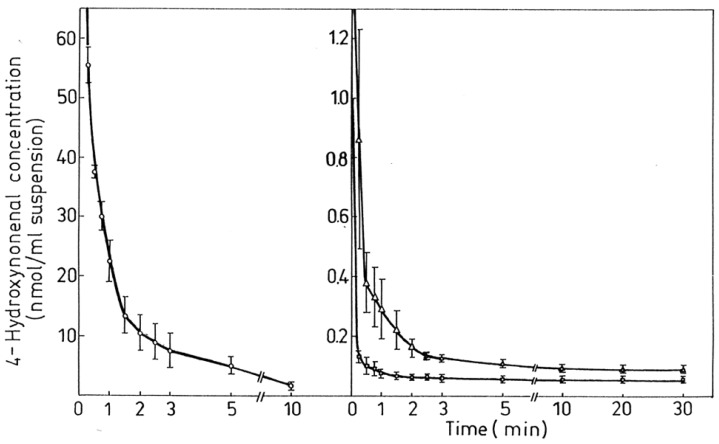
Degradation/metabolism of 4-HNE in rat hepatocytes. The method was the so-called *direct measurement of HNE by HPLC*. The remaining HNE shown in the graphs is the total of not metabolized HNE within the suspension, *i.e.*, intracellular plus extracellular ones at the indicated time-points. The differenciation between the intracellular and extracellular compartments of HNE is not necesssary for balancing the HNE degradation/metabolism. The experimental conditions were: 37 °C, pH 7.4, 1 × 10^6^ hepatocytes/mL suspension; HNE was added at 100 µM (o), 5 µM (∆) or 1 µM (□). Values are the mean ± S.D.

HNE is rapidly metabolized in cells. The velocity and the pattern of HNE metabolism were studied in various cell types, in subcellular organelles and in whole organisms. In all cases the overall metabolic rate of exogenously added HNE was so high, that already within a few minutes equilibrium concentrations in the nanomolar range were obtained (Figure 6). In Table 2 the maximal velocity of HNE total degradation in different biological systems is compared. From these observations it is obvious, that HNE—even at very high lipid peroxidation rates—hardly can accumulate to high levels in biological systems. The highest level ever measured to our knowledge was 6.5 μM (besides the 50 µM in monocytes fed with hemozoin). This value of 6.5 µM was recorded in experiments with rat intestine during the postischemic reperfusion period [266].

In all experimental models investigated a variety of HNE metabolites was identified and quantified. However, one may differentiate these metabolites into primary and secondary intermediates. The primary metabolites are undergoing further metabolic conversion leading to secondary intermediates. Some of the secondary intermediates are stable end products. Possibly one may use such stable HNE products—such as mercapturic acids excreted in urine—as biomarkers for oxidative stress, specifically for LPO, also in medical diagnostics. Additionally, the HNE metabolism is an important component of the antioxidative defense system of cells because of the cytotoxic, mutagenic, and even carcinogenic effects of aldehydic products of LPO, namely of HNE.

**Table 2:** Maximal velocity of total HNE degradation in cells, subcellular organelles, and perfused organs.

Biological System	Maximal Rate of HNE Catabolism	Reference
Kidney cortex mitochondria, rat	112.2 nmol/mg protein/min	[292]
Hepatocytes, rat	28.4 nmol/mg w.w./min	[293]
Ascites tumor cells	9 nmol/mg w.w./min	[264]
Thymocytes, mouse	27.7 nmol/mg w.w./min	[294]
Synovial fibroblasts, rabbit	27.3 nmol/10^6^ cells/min	[295]
Perfused kidney, rat	160–190 nmol/g w.w./min	[296]
Perfused intestine, rat	22 nmol/g w.w./min	[266]
Perfused heart, rat	50 nmol/g w.w./min	[264]

The velocity of HNE metabolism was often measured by tracer kinetic methods. Mostly [2-3H]-HNE was used [266,292,293,297,298]. Sometimes only the so-called “direct HNE measurement” (free or unbound HNE) by HPLC was used detecting the release of added HNE [263,292]. In other studies, namely those investigating specific parts of HNE catabolism, glutathione consumption after adding different HNE amounts was analysed [299]. HNE added to hepatocyte suspensions (10^6^ cells/mL) in concentrations of 1, 5, or even 100 μM was almost completely metabolized within three minutes [300,301,302,303] (Figure 6).

Originally it was assumed that HNE metabolism is especially fast in hepatocytes [300,304,305,306] because the first studies on cellular HNE metabolism demonstrated that compared with liver, all other tissues possess only a low capacity to metabolize HNE, ranging from 0% (fat pads) to 10% (kidney) of the activity present in liver [300]. However, later on also in other cells types a rapid HNE metabolism was found, in some cell types such as thymocytes even in a similar range as in hepatocytes. Furthermore, the first findings on the metabolism of HNE by isolated hepatocytes and by liver homogenate cytosolic fractions suggested that hepatocytes and rat liver cytosol, respectively convert HNE enzymatically mainly to the corresponding alcohol, dihydroxynonene (DHN; non-2-ene-1,4-diol) via alcohol dehydrogenase. It was therefore postulated that the rapid conversion of the cytotoxic HNE and other reactive aldehydes to alcohols, which are probably less toxic, could play a role in the defense system of the liver against toxic substances arising from free radical-induced LPO [300]. This view was expanded later after the identification of a multitude of HNE products with quantitative majorities of the GSH-adduct(s) and/or hydroxynonenoic acid (HNA) and its further degradation products rather than a majority of the alcohol DHN [293].

### 7.1. HNE Metabolism in Mammalian Cells and Organs

HNE metabolism was studied in hepatocytes [293,300,302,306], hepatoma cells [307,308,309,310], ascites tumor cells [264], mucosal cells [297], synovial fibroblasts [295], thymocytes [294], vascular smooth muscle cells [311] and also in organs such as heart [264,312] and kidney [296,298]. The rapid HNE degradation found and the intracellular metabolism implicate that HNE can rapidly enter cells. The velocity of HNE entry into the cells was measured by tracer kinetics in various cell types [295]. In ascites tumor cells, the total rate of HNE metabolism by cells in the proliferating phase (early phase) and cells in the stationary phase (late phase or resting phase of tumor growth) was almost the same [264]. The formation of glutathione conjugates following the addition of HNE was higher in early phase cells when compared with cells in the late phase of tumor growth. This observation was in accordance with the increased consumption of the reduced form of glutathione (GSH). The glutathione transferase activity in tumor cells of both proliferation stages was equal, but the substrate GSH had a higher level in cells of the proliferating phase than in the cells of the resting phase. The result was a higher glutathione-adduct formation in cells of the proliferating phase. The reductive pathway of HNE metabolism was high in proliferating phase tumor cells. In contrast, the oxidative pathway was overwhelming in resting phase tumor cells. This fact corresponded with the different energetic and redox state of both phases. The cells of the resting phase showed a higher rate of uncoupling than cells during the proliferating phase, in accordance with a lower NADH/NAD^+^ and lactate/pyruvate ratio of resting cells. The consequence of the high demand of reduced NADH by partially uncoupled mitochondria for ATP production is a lower capacity of resting cells to use NADH for HNE reduction (DHN or GSH-DHN formation). In support, addition of NADH to the homogenates of rat hepatoma cells (MH1C1) resulted in a 1.5-fold increase of aldehyde consumption [309].

In hepatoma cells, such as MH1C1 and HTC, the metabolic pathway for HNE removal is markedly different from hepatocytes. The results of several investigations indicate that the liver biochemical pathways involved in HNE metabolism undergo a complex variety of changes during neoplastic transformation, so that hepatoma cells with different degrees of deviation show different metabolic patterns with respect to HNE. The analysis of aldehyde enzymatic activities for example showed that MH1C1 cells have decreased levels of all enzymes involved in the catabolism of HNE except for NAD and NADP aldehyde dehydrogenase in the cytosol, compared to hepatocytes and to HTC cells. HNE can, however, be metabolized through a sufficiently high activity of glutathione S-transferase. NAD and NADP dependent aldehyde dehydrogenases are of great importance in HTC cells for HNE metabolism. In HTC cells the metabolic efficiency is due mainly to oxidative enzymes [310].

Comparing Chinese hamster fibroblast control cells (HA-1) with Chinese hamster fibroblast H_2_O_2_-resistant cell lines (OC5 and OC14) the H_2_O_2_-resistant cells were found to be significantly more resistant than HA-1 cells to the cytotoxicity of HNE, as determined by clonogenic cell survival. This finding was related to a significant 2–3-fold increase in the HNE degradation rate of OC5 and OC14 cells, *i.e.*, the HNE removal from culture media containing 72 μM HNE when compared with HA-1 cells [313]. Thus, the resistance appeared to be related to increased cellular HNE metabolism.

### 7.2. HNE Metabolism in Subcellular Organelles

HNE metabolism was studied in several mitochondrial suspensions. By analyzing the decrease of the HNE concentration in the first 30 s after treatment with 100 μM HNE, Ullrich *et al.*, 1994 calculated the maximal HNE detoxification rate in rat kidney cortex mitochondria to be 112.2 nmol/mg protein/min [292]. After addition of 1 μM HNE the degradation of HNE approached a steady state after 2 min. The steady state for the remaining HNE level has been adjusted to about 0.1 μM, a range which is also known as the steady state value in human blood plasma.

HNE is metabolized by renal mitochondria to 4-hydroxynonenoic acid (HNA), 1,4-dihydroxynonene (DHN), and the GSH-HNE-adduct (GSH-HNE). GSH-HNE and HNA reached the maximum value about 2 min after HNE addition to mitochondria, and thereafter these metabolites slowly decreased. Almost 10% of the radioactivity was bound to proteins. The protein bound radioactive HNE was found predominantly bound to the proteins of the intermembrane space. Lower amounts were bound to the proteins of the inner membrane and to the proteins of the matrix. The nmol HNE value per mg protein was found to be very high in the intermembrane space and almost similar for outer and inner membrane and matrix proteins [292]. In rat brain mitochondria it was found, that *in situ* mitochondrial HNE detoxification is affected by decrements in NAD^+^ availability and complex I activity [314]. In investigations of HNE metabolism during diethyl-nitrosamine-induced carcinogenesis in rat liver it was established that the NAD- and NADP-dependent aldehyde dehydrogenases of the cytosolic fraction and the NADP-dependent aldehyde dehydrogenase of the microsomes show higher values in nodules and hepatoma than in normal liver [315]. Both aldehyde dehydrogenase and glutathione S-transferase activities of rat liver mitochondria are reduced in aged animals. Mitochondrial HNE oxidation by aldehyde dehydrogenase and glutathione conjugation of HNE decline at 18 and 24 months, respectively, while these enzyme activities were found to be well-preserved in dietary restriction animals throughout their life span. These findings indicate that the prevention of the age-associated decrease in HNE detoxification by dietary restriction may be an important mechanism underlying enhanced aldehyde elimination, thus minimizing the functional deterioration observed in mitochondria of old animals [316].

After exposure to racemic HNE, rat brain mitochondria metabolized HNE enantioselectively with a higher rate of (R)-HNE metabolism [317]. By using purified enantiomers of HNE the authors were able to demonstrate that this enantioselective metabolism of HNE was the result of higher rates of enzymatic oxidation of (R)-HNE by aldehyde dehydrogenases compared to (S)-HNE. Conjugation of HNE to glutathione was a minor metabolic pathway and was not enantioselective. These studies demonstrate that the chirality of HNE affects its mitochondrial metabolism.

### 7.3. HNE Metabolism in Whole Animals and Interorgan Relationships

The aim of many *in vivo* metabolic studies was the characterisation of end-products of HNE, especially in urine, in order to develop specific and non-invasive biomarkers of lipid peroxidation. When HNE is administered intravenously, it is mainly excreted into urine and bile as conjugated metabolites, in a proportion that is dependent on the administration route [118,318]. However, biliary metabolites undergo an enterohepatic cycle that limits the final excretion of faecal metabolites. Only a very low amount of metabolites is found to be bound to macromolecules.

According to Alary *et al.*, [118,318] the main urinary metabolites are represented by two groups of compounds. The first group derives from (a) the mercapturic acid formation from 1,4 dihydroxynonene-glutathione (DHN-GSH); (b) the lactone of 4-hydroxynonanoic-GSH (HNA-lactone-GSH) and (c) HNA-GSH. The second group of metabolites is derived from the ω-hydroxylation of HNA or HNA-lactone by cytochromes P450 4A, followed eventually, in the case of ω-oxidized-HNA-lactone, by conjugation with GSH and subsequent mercapturic acid formation. Biliary metabolites are GSH or mercapturic acid conjugates of DHN, HNE and HNA [118,318]. Stereochemical aspects of HNE metabolism are also discussed by Alary *et al.* [118,318]. Experiments with injection of tritium-labeled HNE [319] and also of tritium-labeled 4-hydroxyhexenal (HHE) [320] into the vein of rats have shown that part of the radioactivity was excreted in the urine as mercapturic acid conjugates. From these experiments and studies with perfused kidneys [296] it may be concluded that the excretion of mercapturic acid conjugates as well as the excretion of DHN is the main route of HNE-product disposal from the organism. The most important studies on urinary excretion of HNE metabolites were carried out by the group of Alary, Cravedy, and Gueraud. Following intravenous administration of [^3^H]HNE into rats, the majority of the dose appeared in urine (67.1% after 48 h) [319]. The radio-HPLC metabolic profile showed that no unchanged parent compound was detected in urine whereas at least four metabolites were present, most of them corresponding to mercapturic acid conjugates.

Two major pathways were involved in the biotransformation of HNE *in vivo*: the reduction/oxidation of the aldehyde group, and the conjugation to endogenous glutathione leading to mercapturic acid conjugates in urine. These end products were isolated by HPLC and identified by mass spectrometry as HNE mercapturic acid, 1,4-dihydroxynonene mercapturic acid, 4-hydroxynonenoic mercapturic acid, and the corresponding lactone.

### 7.4. Primary HNE Intermediates—Enzymatic Reactions and Quantitative Results

**Figure 7:**
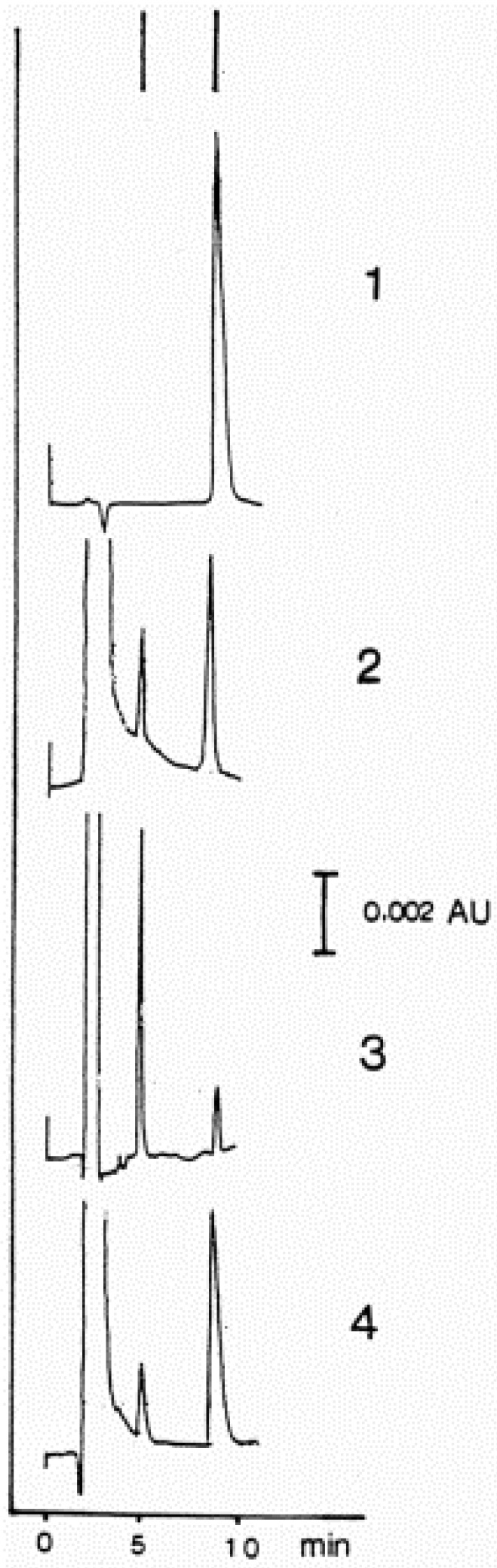
Identification of HNE and 4-hydroxynonenoic acid (HNA) by isocratic HPLC separation. Conditions: the eluent was 42% acetonitrile/water v/v 1 mL/min; columns were Spherisorb S5 ODS2 250 × 4.5 mm; detection of both substances at 223 nm; authentic standard of 4-HNA was produced in the system of HNE, dehydrogenase (NAD^+^) and aldehyde dehydrogenase; (**1**) HNE standard solution 30 µM; (**2**) product of oxidation of HNE: HNA 15 µM; (**3**) mixture from cellular incubation mit CH_2_Cl_2_ extract of HNE oxidation; (**4**) mixture formed in rat liver cells (10^6^ cells/mL) after addition of 100 µM HNE, the first peak representing HNA, the second peak representing HNE; for exact quantification of HNA the comparison of the HNA-peak with NADP formation in experiments on transition of HNE by means of aldehyde dehydrogenase was carried out.

**Figure 8:**
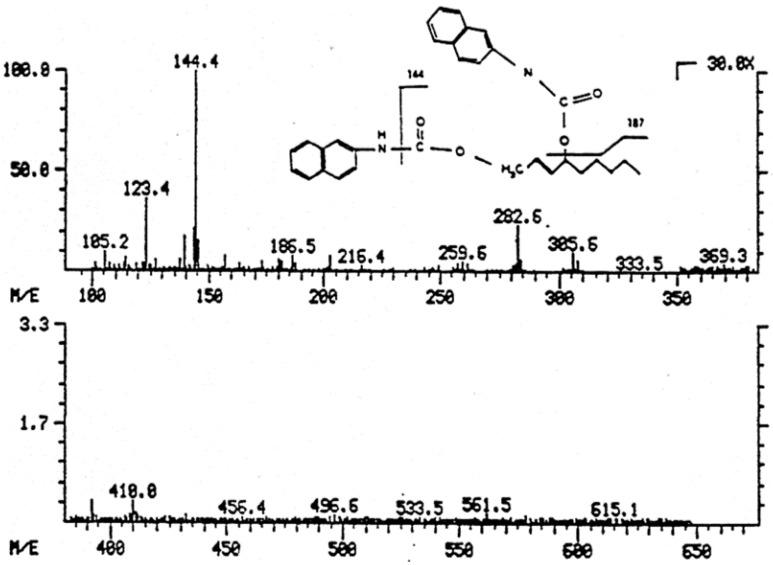
Mass spectrum of dihydroxynonene urethane (HPLC plus MS) with fluorimetric detection. The preparation of the reduced HNE product 1,4-DHN was carried out in the following manner: reduction of HNE with sodium boronate; extraction with dichloromethane; drying by means of sodium sulphate; purification by TLC separation, detection with 10% phosphomolybdenic acid dissolved in ethanol, extraction from TLC plates with CH_2_Cl_2_. Identification of 1,4-DHN: Siliylation; GC (85 °C, with 10 °/min up to 150 °C, 50 m SE30 column—i.d. 0.32 mm, FID); formation of urethane derivative (1,4-dinaphthyl-2,3-nonenylurethane); (305, 350 nm). Preparation of radioactively labelled 1,4-DHN: Reduction of radioactively labelled HNE with sodium boronate; extraction with dichloromethane; purification by means of TLC and elution on Bond Elut C18 columns (35% methanol/water, v/v). Chemical ionization (CH_4_). Peaks: m/e 144 Ar-NH_3_^+^, m/e 187 Ar-NH-COOH, m/e 123 (M + 1)-(2 × 187); (M + 1) = 497 m/e [301].

**Figure 9:**
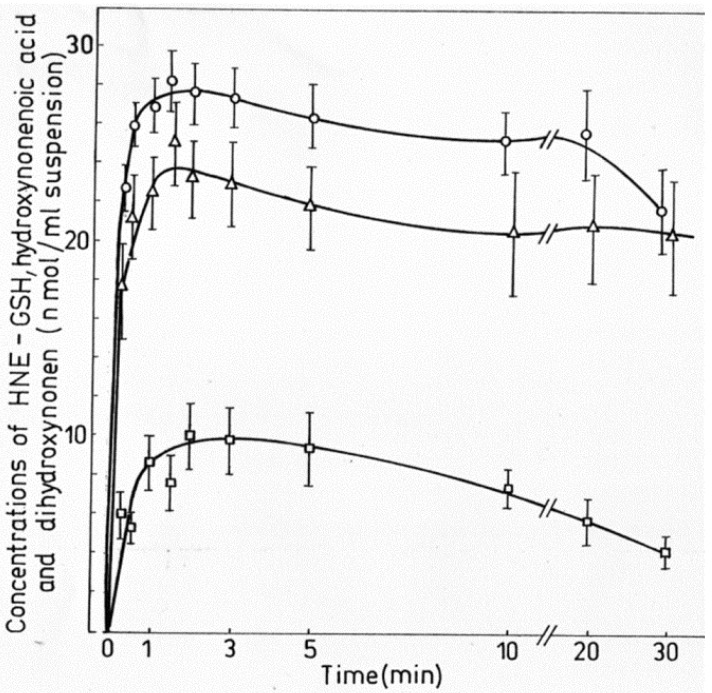
HNE metabolites. Formation of the so-called primary HNE metabolites HNA (∆), HNE-GSH (o) and DHN (□) in rat hepatocyte suspensions after loading the cell suspensions with 100 µM tritium-labelled HNE. The glutathione (GSH) adduct of HNE was quantified as follows: Measurement as isoindol derivative following the reaction of the adduct with o-phthaldialdehyde in the presence of mercaptoethanol; HPLC separation of the isoindol derivative by isocratic HPLC with 65% methanol/0.1 M sodium acetate buffer pH 6.5 (v/v) in the presence of an ion-pair reagent (tetraethyl-ammoniumhydroxide/TAA); fluorimetric detection at 343 and 445 nm; columns: ODS 5 µm, 250 mm × 4.6 mm i.d.; purification of the extracts before HPLC separation on bond Elut C18 columns (methanol, water, sodium acetate buffer, hexane, methanol elutions) [293].

Already in 1991 in the classical HNE-review of Esterbauer *et al.* [304] it was mentioned: the main enzymes involved in HNE metabolism are glutathione transferases, aldehyde dehydrogenases, and alcohol dehydrogenases. These are the enzymes forming the so-called primary HNE metabolites. The primary metabolites of HNE are therefore, the HNE-GSH adduct, the corresponding carboxylic acid of HNE, *i.e.*, HNA, and the corresponding alcohol, *i.e.*, DHN. Figure 7, Figure 8 and Figure 9 provides examples for the analysis of HNE and HNA by HPLC (Figure 7), 1,4-DHN by HPLC plus MS with fluorimetric detection (Figure 8), and formation of HNA, HNE-GSH and DHN in hepatocytes (Figure 9). As displayed in Figure 9, after addition of a single dose of HNE to a hepatocyte suspension all three primary HNE metabolites increased very rapidly within the first minute of the experiment and reached a maximum value—in case of 100 μM HNE—after about 2 min, and thereafter slowly decreased. At all time points the GSH-HNE conjugate was the main product of hepatocytes followed by HNA. The concentration of DHN was considerably lower. The relative proportion of the three metabolites in hepatocytes was approximately GSH-HNE:HNA:DHN = 6:4:1, a pattern which may be quite different in other cell types. The formation of the GSH-HNE conjugate always leads to a decrease of intracellular GSH concentration. The sum of the three primary metabolites, GSH-HNE + HNA + DHN, in hepatocytes after 3 min of incubation ranged between 60% and 65% of the added HNE.

Taking into account the small fraction of the non-metabolized HNE, it follows that about 33% to 36% of the HNE was converted into other metabolites. This fraction involves secondary metabolites and HNE-modified proteins and peptides. In hepatocytes, this fraction was below 10% within the very first initial phase of about 30 s of incubation after addition of exogenous HNE. With increasing duration of the incubation this fraction steadily increased—while the sum of GSH-HNE, HNA, and DHN decreased again—and reached a value of nearly 50% after 30 min. These findings strongly suggest that the primary metabolites GSH-HNE conjugate, HNA, and DHN are further metabolized to secondary products [293].

As mentioned, the metabolism of HNE involves multiple pathways, including conjugation with glutathione catalyzed by glutathione S-transferases (GST) [321,322], oxidation of the aldehyde functional group to form HNA catalyzed by aldehyde dehydrogenases [323,324], and reduction by alcohol dehydrogenase to 1,4-dihydroxynonene [300]. The HNE-metabolizing aldehyde dehydrogenase isoenzymes are present in the hepatic cytosol, mitochondria, and probably also in microsomes [303,325]. The HNE-metabolizing NADH-dependent alcohol dehydrogenase is localized mainly in the hepatic cytosol [303]. The glutathione transferases are ubiquitous enzymes, being particularly rich in hepatocytes and probably other liver cells. Even though a number of rat GST isozymes show catalytic activity toward HNE, rat liver glutathione transferase 8-8 has been demonstrated to be most effective in catalyzing the conjugation of this compound to GSH, and it has been suggested that this isozyme may be involved in the detoxification of HNE and related endogenous electrophiles [303,304,321,322].

A human acidic glutathione-S-transferase, hGST 5.8, was isolated from liver, heart, bladder, pancreas, and brain [326], and it was shown that this enzyme has an about 20-fold higher specific activity for HNE than for 1-chloro-2,4-dinitrobenzene, and expressed glutathione peroxidase activity toward phospholipid hydroperoxides. The hGST 5.8 was absent in lung, kidney, skeletal muscle, colon and erythrocytes [326]. Berhane *et al.*, 1994 characterized the detoxification of base propenals (degradation products of DNA) and other unsaturated aldehyde products of free radical reactions and lipid peroxidation by human glutathione transferases [327]. They found that in general, GST A1-1 and GST M1-1, in contrast to GST P1-1, were more active with 4-hydroxyalkenals than with base propenals. HNE readily reacts with GSH also in a nonenzymatic reaction. The GSH transferase-catalyzed reaction can, however, proceed about 300 to 600 times faster [304].

GST can be induced by HNE. To investigate the effect of LPO products on the expression of GSTs Fukuda *et al.* [328] exposed normal rat liver epithelial cells (RL34) to aldehydic compounds and found out that GST activity was induced by α,β-unsaturated aldehydes, such as acrolein (1.3-fold), crotonaldehyde (1.4-fold), 4-hydroxyhexenal (HHE) (1.4-fold), and HNE 1.7-fold. The induction of GST activity by HNE was time-dependent, reaching a plateau after 16 h. The authors concluded that the induction of GSTs by HNE may represent an important cellular defense mechanism against oxidative injury [328]. Immunoblot analysis applying polyclonal antibodies against GST isozymes demonstrated that GST-P, a well-known tumor marker, was significantly induced 16 h after HNE treatment [328]. In early human fibrous atherosclerotic plaques, immunohistochemical studies demonstrated marked induction of hGST A4-4 in endothelial cells overlying plaque, and in proliferating plaque vascular smooth muscle cells [329]. The authors conclude that endothelial cell mGST A4-4 can play a key role in protecting blood vessels against oxidative stress and, thus, is likely to be a critical defense mechanism against oxidants that act as atherogens. In rat aortic smooth muscle A10 cells the induction of GSH and glutathione S-transferase by 3H-1,2-dithiole-3-thione (D3T, a chemoprotective chemical known to induce detoxification enzymes), and protective effects of D3T, *i.e.*, elevated cellular defenses against HNE-mediated toxicity were characterized [330]. As results the authors report that incubation of A10 cells with D3T resulted in a marked concentration-dependent induction of both GSH and GST, that this induction exhibited a time-dependent response, and that pretreatment of A10 cells with D3T led to a dramatic decrease of HNE-induced cytotoxicity.

A redox activation of aldose reductase (AR) in the ischemic heart was shown by Kaiserova *et al.*, 2006 [331]. Their data indicate that reactive oxygen species formed in the ischemic heart activate AR by modifying its cysteine residues to sulfenic acids. To investigate the role of AR in the late phase of ischemic preconditioning, Shinmura *et al.*, 2002 treated conscious rabbits with six cycles of 4-min coronary occlusion/4-min reperfusion [332]. 24 h later they observed s a marked increase in AR protein and activity and in the myocardial content of sorbitol, a unique product of AR catalysis. AR was demonstrated to be an essential mediator of late preconditioning. Furthermore, the data suggest that myocardial ischemia/reperfusion injury is due in part to the generation of LPO products and that late preconditioning diminishes this source of injury by upregulating aldose reductase [332].

Aldose reductase (AR), an NADPH-dependent aldo-keto reductase, which catalyzes the reduction of glucose to sorbitol as part of the polyol pathway, is able to catalyze the reduction of a very broad range of aldehydes. HNE is an excellent substrate (Km = 22 μM, Kcat/Km = 4.6 × 10^6^ M^−1^·min^−1^) [332]. Therefore, it was concluded that the extrahepatic reductive metabolism of HNE involves AR [332]. ARs, present in human kidney and other tissues play an important role in the reductive metabolism of endogenous aldehydes such as HNE [333]. The kidney contributes via this pathway to a rapid urinary excretion of HNE products from the organism. It is worth to mention that AR is the only major HNE oxidoreductase of the human heart. AR has been purified from bovine retina, too. It has an apparent molecular weight of 32 kilodaltons and shares immunological and kinetic properties with the much studied aldose reductases purified from various sources. Retinal aldose reductase displays a Km of approximately 40 μM for HNE and 4-hydroxyhexenal. Thus, AR was suggested to constitute a major detoxification route for these toxic aldehydes also in the retina [334]. Inhibition of aldose reductase by fidarestat suppresses several oxidative stress-induced inflammatory disorders. Fidarestat has already undergone phase III clinical trial for diabetic neuropathy and was found to be safe, though clinically not very effective [335]. It was also shown, that HNE and MDA induce the expression of the aldehyde reductase gene, which could be prevented by the carbonyl scavenger *N*-acetyl-l-cysteine (NAC) [336].

In other experiments effects of HNE addition on cellular glutathione levels were analyzed [299]. The authors studied the dose-dependent cytotoxicity of HNE after treatment of cultured fibroblasts at doses up to 50 nmol/10^6^ cells for 3 h. In peroxide resistant OC14 and oxygen resistant O2R95 cells variants of HA1 cells, HNE treatment resulted in a statistically significant consumption of GSH at all doses tested. Cellular GSH levels in HA1 and O2R95 cells fell—starting from about 25 nmol GSH/mg cell protein—below 5 nmol GSH/mg cell protein at HNE doses of 25 and 40 nmol/10^6^ cells, respectively. That argues for a very high rate of GSH-HNE adduct formation. In OC14 cells, GSH consumption—starting from about 35 nmol/mg protein—could not be detected until HNE doses exceeded 50 nmol/10^6^ cells and did not fall below 5 nmol GSH/mg cell protein at any dose tested. In OC14 cells obviously the GSH-HNE adduct formation rate is very low [299]. This cannot be due to the capacity of glutathione-S-transferase which was even higher in OC14 cells compared to HA1 and O2R95 cells and stayed stable during the 3 h of HNE treatment in all three cell types, but rather to the enzymatic properties which obviously differ from one cell type to the other. These experiments also support the high stability of glutathione-S-transferase in these cultured fibroblast cell lines.

Table 3 displays the formation of primary HNE metabolites for different cells and tissues after the addition of 100 μM HNE to the biological system.

**Table 3:** Primary HNE metabolites in different cells and tissues after the addition of 100 μM HNE to the biological system.

Biological System	DHN	GSH-HNE	HNA	HNE-P	Reference
Kidney mitochondria (rat)	9.3 ± 1.8	9.8 ± 1.2	5.9 ± 0.7	8.4 ± 1.4	[292]
Hepatocytes (rat)	8.1 ± 2.1	27.5 ± 2.5	25.3 ± 5.6	2.0 ± 0.6	[293]
Enterocytes (rat)	5.4 ± 0.6	11.0 ± 0.5	4.2 ± 0.6	1.3 ± 0.2	[297]
Thymocytes (mouse)	6.9	10.2	6.8	2.8	[294]
Synovial fibroblasts (rabbit)	0.3 ± 0.1	29.1 ± 1.9	1.0 ± 0.3	4.3 ± 0.5	[295]
Kidney venous effluent (rat)	24 ± 5	14 ± 4	5 ± 1	n.d.	[296]
Ehrlich ascites cells, proliferating	2.0	10.5	3.0	7.2	[264]
Ehrlich ascites cells, resting	1.5.	8.0	3.8	1.5	

HNE-P, HNE-protein; n.d., not determined. Given values represent the % of the initial HNE concentration (mean; mean ± S.D.).

### 7.5. Secondary HNE Intermediates—Enzymatic Reactions and Quantitative Results

It is known from many studies that HNE can bind to thiol-groups of proteins. In general it is well established that HNE forms adducts with the following side chains in proteins: Cys, His and Lys. From tracer-kinetic experiments it was estimated that between 1% and 8% of exogenously added or endogenously formed HNE reacts with proteins to form HNE-protein conjugates. With that share of the totally added or formed HNE the proteins and peptides are the most important group of biomolecules targeted by HNE. In experiments studying the metabolism of exogenously added HNE always a dynamic pattern was seen: the binding of labelled HNE in the HNE-protein conjugates-pool reached a maximum—in hepatocytes 3 min after HNE addition—followed by a decline, underlining the fact that aldehyde-modified proteins underlie a removal of oxidatively damaged and modified proteins by the proteasomal system [337,338,339]. Nevertheless, the formation of HNE-protein and HNE-peptide compounds does not represent a metabolic pathway of HNE degradation. The chemical reactions leading to HNE modifications of proteins, peptides, and further biomolecules rather reflect this part of HNE “removal” which leads to damaging or signal effects by HNE. The real metabolic reactions of HNE lead to the primary and secondary HNE metabolites, which finally contribute to the detoxification and harmless removal of HNE.

*Which are the real secondary metabolic products of HNE?* The pool of secondary HNE intermediates contains degradation products of HNA, which is degraded via different products of β-oxidation to acetyl-CoA and, finally to carbon dioxide and water. This involves the reaction steps of the tricarboxylic acid cycle and of the respiratory chain. In experiments with ^3^H-labeled HNE the formation of ^3^H-labeled water could be shown in hepatocytes [293]. The rate of formation of radioactive water was slow, but the time curve showed not a transient maximum but a steady increase. In hepatocytes treated for 10 min with 100 μM HNE, 14% of the radioactivity appeared in the water peak. The formation of 3H-labeled water could be almost completely inhibited in presence of 4-pentenoic acid, an inhibitor of β-oxidation. In the presence of 4-pentenoic acid only traces of radioactive water were formed.

Furthermore, in the presence of this inhibitor there was a significantly higher accumulation of 4-hydroxynonenoic acid (HNA) in comparison with control experiments without inhibitor. In the presence of 4-pentenoic acid HNA became the major HNE metabolite. During the first 60 s of incubation, the inhibitor had almost no effect on the rate of the conversion of HNE to HNA. Thereafter, however, the time profile was markedly different. In the presence of the inhibitor, HNA steadily increased, reaching 45 nmol/mL after 3 min, whereas in the absence of the inhibitor the concentration of the carboxylic acid slowly decreased again. These experiments indicate that HNA is not an end product of HNE metabolism, but is further metabolized [293].

The oxidative pathway includes—when HNA is degraded—also tricarboxylic acid cycle products, such as aconitate, citrate, malate, fumarate, *etc.* In primary cultures of rabbit synovial fibroblasts 3 min after addition of 100 μM labelled HNE the shares of radioactivity of tricarboxylic acid cycle intermediates and of water were 7.4% and 34.1%, respectively [295]. The β-oxidation of HNA including the formation of tritiated water was also demonstrated in liver slices [340]. The identification of 4-hydroxy-9-carboxy-2-nonenoic acid and 4,9-dihydroxynonanoic acid demonstrated that ω-oxidation significantly contributes to the biotransformation of HNE in liver slices [340]. Alary *et al.*, 1998 demonstrated the existence of further secondary products generated from HNA. These are the ω-oxidation product of HNA, namely 9-hydroxy-HNA, and the subsequent oxidation product of 9-OH-HNA, 9-carboxy-HNA [341]. Furthermore, 9-hydroxy-DHN was identified as ω-hydroxylated product of DHN [341].

The sequence of β- and α-oxidations in the pathway from HNA-CoA to the citric acid cycle was described in detail by Li *et al.*, 2013 [342]. They showed that after the formation of HNA-CoA a β-oxidation takes place leading to the formation of 2-hydroxy-heptanoyl-CoA. This intermediate is converted by α-oxidation to hexanoyl-CoA, and by three additional β-oxidations to acetyl-CoA. HNA-CoA can alternatively be phosphorylated to give 4-phospho-nonanoyl-CoA. The latter is hydrolyzed to 3-hydroxy nonanoyl-CoA, which is converted by three β-oxidations to propionyl-CoA, then to methylmalonyl-CoA and finally to succinyl-CoA as intermediate of the citric acid cycle [342].

Another group of secondary intermediates which was identified and quantified are the GSH-DHN and GSH-HNA conjugates as additional GSH-conjugates besides GSH-HNE [118]. Mercapturic acids were demonstrated to be the final products of this part of HNE metabolism (see also Chapter 6.2.). Different further secondary HNE products were postulated, such as 1-hydroxy-4-oxononene and others. In astrocytic biotransformation of HNE two novel metabolites of HNE were discovered, gamma-nonalactone and the potent pyrrole forming compound, 4-oxo-nonanal [343].

Compared to other organs, liver and kidney seem to be the most important organs for the elimination of the final products of metabolism. The importance of the kidney in the formation of HNE-mercapturic acid conjugates was demonstrated by Alary *et al.*, 1995 [319] and Grune *et al.*, 1997 [296]. HNE mercapturic acid, 1,4-dihydroxynonene mercapturic acid, 4-hydroxynonenoic mercapturic acid, and the corresponding lactone as HNE intermediates in urinary excretion were already mentioned above [319]. In studies with perfused kidneys it was shown, that all metabolites of HNE are also found in the urine. HNA, the HNE-mercapturic acid conjugates, and DHN had a higher concentration in urine than in the venous effluent of the kidney [296]. When 100 μM HNE was used for perfusion the highest HNE metabolite concentrations were found for DHN with 53 nmol/mL in urine and 24 nmol/mL in the venous effluent. The GSH-HNE conjugate and the products of the TCA-cycle never reached concentrations above 5 nmol/mL in urine. In the venous effluent their concentrations were about 15–17 nmol/mL which was 4–6-fold higher than in urine. HNE-mercapturic acids seem to be stable products of HNE metabolism which are excreted from the organism. 1,4-dihydroxynonene mercapturic acid (DHN-MA), was shown by Alary *et al.*, 1998 to be the major urinary metabolite of HNE administered to the rat [344]. It was characterized and determined to be a normal constituent of both rat and human urine. DHN-MA was excreted as a mixture of at least two stereoisomers. The 24-h urinary excretion of this compound was about 10 ng and 5 µg for rat and human, respectively. Following i.v. administration of HNE and [4-^3^H]HNE to rats, besides DHN-MA 15 polar urinary metabolites accounting for about 50% of the urinary radioactivity were separated by HPLC by the same group [341]. Among them, eight major compounds and tritiated water were quantified. Most of polar metabolites were found to originate from ω-oxidation of 4-hydroxy-2-nonenoic acid (HNA) to 9-hydroxy-HNA, its mercapturic acid conjugate, and two diastereoisomers of the corresponding lactone. 9-Hydroxy-HNA is a substrate for alcohol and aldehyde dehydrogenases leading to the formation and excretion of 9-carboxy-HNA and of the corresponding lactone mercapturic acid conjugate. The 9-hydoxy-HNA and the 9-carboxy-HNA were already mentioned above. The alcohol dehydrogenase metabolite 1,4-dihydroxy-2-nonene (DHN) was to a lower extent ω-hydroxylated, leading to 9-hydroxy-DHN which was found to be excreted as a mercapturic acid conjugate [341].

To characterize the metabolic pathways involved in the formation of urinary HNE-MA conjugates in the rat, the metabolism of HNE-thioethers HNE-GSH, HNE-MA, and HNE-Cys by rat liver and kidney cytosolic fractions was investigated by Alary *et al.*, 2003 [318]. The results showed that HNE-GSH is a good substrate for cytosolic incubations whereas HNE-MA and HNE-Cys are poorly metabolized. About 80% of the urinary MA conjugates originate from the primary and major HNE metabolite, namely, the hemiacetalized HNE-GSH. The direct reduction of HNE-GSH by a cytosolic aldo-keto reductase (NADPH) leads to DHN-GSH and subsequently to DHN-MA. The direct oxidation of HNE-GSH by aldehyde dehydrogenase (NAD^+^) leads to 4-hydroxynonenoic-lactone-GSH, the partial hydrolysis of which occurs at physiological pH and accounts for the corresponding HNA-GSH. Both the spontaneous- and the glutathione S-transferases-catalyzed retro-Michael cleavages of HNE-GSH and HNA-lactone-GSH are the source of HNE and HNA-lactone, respectively. HNA-lactone with both lipophilic and electrophilic properties is available for microsomal ω-hydroxylation by cytochrome P450 4A enzymes and conjugation with thiol groups, and therefore is the most likely candidate for the formation of ω-hydroxylated HNE-MA conjugates excreted in rat urine [318].

HNE biotransformation products were also detected in the bile of rats following a single intravenous administration of [4-(3)H]HNE. Five metabolites were present in the bile, two of them corresponded to GSH-HNE and to GSH-DHN adducts. Two further metabolites were identified as DHN and HNA-lactone mercapturic acid conjugates, whereas the fifth metabolite in the bile remained unidentified [345]. The kinetics of excretion of biliary metabolites demonstrated a rapid metabolism of HNE in rats: Within 4 h of injection, the bile accounted for 19.5% of the injected radioactivity, whereas only 3% was found in the feces within 48 h. All rat recipients were found to have measurable levels of HNE metabolites in bile, confirming that HNE undergoes enterohepatic recirculation, and the extent of recycling was about 8% of the total dose [345]. In this context it has to be mentioned that the multidrug resistance-associated protein2 (MRP2) plays an important role in the biliary excretion of glutathione conjugates of HNE [346].

The metabolism of the two enantiomers of HNE, the (R)- and the (S)-enantiomer of its racemic mixture, was the aim of a study by Gueraud *et al.*, 2005 [29]. The pathways were investigated in male rats after intravenous administration of the corresponding radiolabeled compounds and compared with the racemic mixture. Although the difference in the excretion rates was not statistically significant, the metabolic urinary pattern showed qualitative and quantitative differences. The level of 3-mercapturic acid-1,4-dihydroxynonene, which is considered as the major urinary metabolite of HNE, was significantly lower in the case of (S)-HNE injected rats. In order to clarify the intermediate pathways the authors performed *in vitro* studies using rat liver cytosolic incubations and HNE-glutathione conjugates as substrate and found large differences in the reduction and retro-Michael conversion steps of the metabolism between the conjugates originating from the two enantiomers [29]. Studies on the enantioselective metabolism of HNE by brain mitochondria were already described above [317] (see Chapter 7.2).

#### Determination of HNE Metabolic Products and Pathways

For the determination of HNE intermediates HPLC, HPLC-MS, and TLC were used, sometimes applying radioactively labelled compounds. In tracer kinetic experiments using tritium labelled HNE the HNE metabolites were separated by means of different methods (TLC or HPLC), and the formation of the products was simply calculated using the radioactivity of the spot or peak and dividing it by the specific radioactivity of the added HNE which can be taken as constant taking into account the overshoot of added radioactive HNE in comparison with the “low” formation of new HNE. After addition of labelled HNE aliquots of suspensions were taken. The HNE metabolizing reactions were stopped by the addition of e.g., 0.5 mL suspension to an equal volume of acetonitrile/acetic acid mixture (96:4; v/v). After centrifugation two aliquots of the supernatant were eluted on TLC plates. The elution with hexane/diethylether (3:7; v/v) allows the separation of HNE, HNA, and DHN. The elution with butanol/acetic acid/water (4:1:1; v/v/v) allows the determination of GSH-adducts of HNE [294]. For quantification of HNE utilization rates the radiolabeled compounds have to be scanned by an automatic TLC linear analyzer or to be scraped off and analyzed.

Sometimes acetonitrile/acetic acid extracts were used for the determination of HNE and HNA by an HPLC-method [292,293]. The disappearance of initial HNE levels, such as 100, 10 or 1 μM HNE, can be measured only by an HPLC-method (determination of remaining HNE during the whole experiment). The conditions for this “direct HNE and HNA analysis” are: C18 column, acetonitrile/water (4:6; v/v), 1 mL/min, 223 nm detection wavelength. Quantification of the acid was based on the comparison of the hydroxynonenoic acid peak with formation of NADH in experiments in which HNE reacted with aldehyde dehydrogenase. In those experiments, the rate of formation of NADH, which was observed spectrophotometrically at 340 nm, was equal to the consumption rate of HNE (measured by HPLC at 223 nm) and to the rate of formation of HNA. From the NADH formation rate (equal to the HNE consumption rate) and the peak integral of the HNA produced, one can calculate the extinction coefficient of the acid or the amount of HNA representing a defined peak area in the HPLC separation procedure [293]. HNE quantification was done by means of authentic HNE samples of known concentration, dissolved in methanol-water 80:20 (v/v) and comparison of the peak height of the standard chromatogram to sample chromatograms. A chromatogram of this direct HNE and HNA determination is shown in Figure 9.

**Figure 10:**
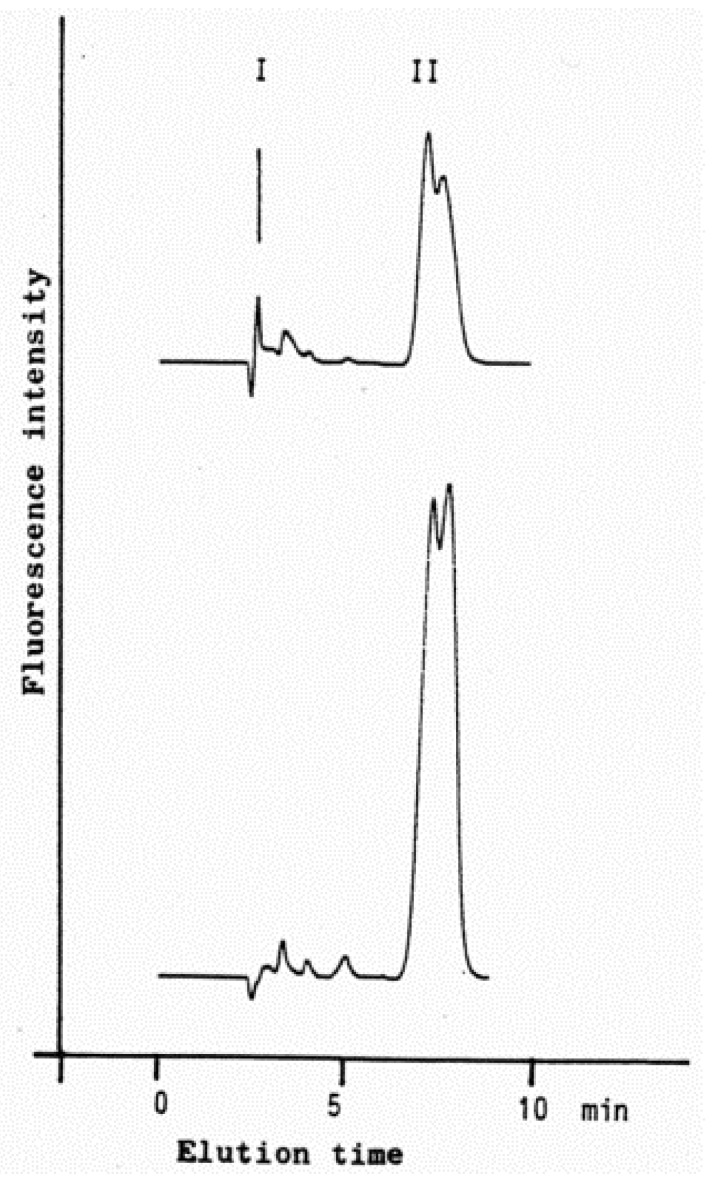
HPLC chromatogram of the isoindol derivative of the HNE-GSH conjugate (reaction product in presence of o-phthalaldehyde). Fluorimetric detection at 345 nm (excitation) and 445 nm (emission). Peaks: I GSH derivative; II HNE-GSH derivative; upper panel: Standard solution (45 µM); lower panel: extract from hepatocyte suspension incubated in presence of 100 µM 4-HNE [301].

The GSH-HNE-adduct was measured as an isoindol derivative after the reaction of the adduct existing in neutralized perchloric acid extracts with o-phthaldialdehyde in the presence of mercaptoethanol (Figure 10).

The reaction is started by addition of a sample aliquot to the o-phthaldiadehyde solution. The reaction mixture is transferred into a Hamilton syringe and the reaction is stopped by injection of the mixture into the HPLC system after 2 min. HPLC separation is carried out in an isocratic manner with 65% methanol-0.1 M sodium acetate buffer, pH 6.5 (v/v) in the presence of an ion-pair reagent (tetraethylammoniumhydroxide). Isoindol is detected by means of a fluorescence detector at wavelengths of 345 nm and 445 nm for excitation and emission, respectively. The formation of enantiomer products in the reaction of GSH-HNE adduct with o-phthaldialdehyde [347] led to the appearance of a double peak of the isoindol adduct (Figure 10). The reaction product of free reduced glutathione (GSH) with o-phthaldialdehyde eluted much earlier than the isoindol adduct within the isocratic HPLC separation. Precise measurement of the adduct concentration requires an extensive preparation of the neutralized perchloric acid extract of suspensions with the aim of removing disturbing influences of amino acid derivatives after HPLC separation. For the purification of the extracts, Bond Elut C18 columns and a Baker-10 Extraction system can be recommended [293]. The columns should be rinsed with methanol and equilibrated with water. After injection of the sample the column should be rinsed with 0.1 M sodium acetate buffer, pH 5, then rinsed with hexane. The sample is then eluted with methanol, evaporated to dryness, and redissolved in a defined volume of methanol [293]. The protein precipitate is washed with physiological saline solution, then dissolved in Protosol-Tissue and Gel Solubilizer and added to toluene scintillation fluid and measured by means of a β-counter if using ^3^H-labeled HNE [292,297].

Different HPLC separations were applied in tracer kinetic experiments and in measurements of non-labelled HNE intermediates [293,312,319]. Urinary end products of HNE such as HNE mercapturic acid, 1,4-dihydroxynonene mercapturic acid, 4-hydroxynonenoic mercapturic acid, and the corresponding lactone were isolated and identified by mass spectrometry [319]. The HPLC-separation used by Srivastava *et al.*, 1998 was combined with different detection methods: electrospray ionization MS for the detection of HNE-GSH and DHN-GSH, GC-chemical ionization MS for HNA [312]. Inhibitors were applied to modify the HPLC peak pattern. Sorbinil, an inhibitor of aldose reductase, attenuated the DHN-GSH peak, and cyanamide, an inhibitor of aldehyde dehydrogenase (ALDH), attenuated HNA formation.

Various enzyme inhibitors were useful in studies on HNE metabolism. In investigations of HNE metabolic pathways of vascular smooth muscle cells it was shown that the formation of HNA was inhibited by cyanamide; indicating that the acid is derived from an ALDH-catalyzed pathway; while the overall rate of HNE metabolism was insensitive to inhibition of aldose reductase or aldehyde dehydrogenase. Nevetheless; inhibition of HNA formation by cyanamide led to a corresponding increase in the fraction of HNE metabolized by the GSH-linked pathway; indicating that ALDH-catalyzed oxidation competes with glutathione conjugation [311].

### 7.6. HNE Metabolism as a Component of the Antioxidative Defense System

The catabolism of HNE may be regarded as a very important part of the antioxidative defense system of cells and organisms. This is due to the fact, that HNE like other aldehydic products of LPO is able to exert cytotoxic, mutagenic, signal, and carcinogenic effects as described above. The degradation of HNE to less toxic intermediates diminished the reactions between HNE and biomolecules and therefore effectively contributes to the antioxidative protection of cells and organisms. This protective effect is the more effective the more rapidly the metabolism of HNE leads to stable HNE products, which can be easily excreted. The primary importance of HNE-degrading pathways which function both at physiological and pathophysiological HNE levels, seems to be the protection of proteins from modification by aldehydic LPO products. This should especially be valid in regions with high HNE formation rate such as in postischemic or reperfused tissues or in synovial tissue of joints during rheumatoid arthritis and other inflammatory diseases or in lymphedematous tissue, *etc.*

### 7.7. HNE Intermediates as Potential Biomarkers of LPO

Two groups of HNE derivatives seem to be useful as possible biomarkers in clinical chemistry and laboratory medicine. The first are the mercapturic acid conjugates including the derivatives of HNE, HNA, and DHN. These conjugates are excreted with the urine and, therefore can be analyzed from urine. The second are HNE-protein adducts of blood serum or plasma. The HNE-modified plasma proteins are already used for evaluation of rheumatic and autoimmune diseases [348]. Both body fluids—urine and serum or plasma—are easily obtained from patients. Both groups of derivatives are relatively stable. While the mercapturic acid assays are time- and equipment-consuming, the determination of HNE-modified plasma proteins is quite easy to handle. Within the mercapturic acid conjugates Peiro *et al.*, 2005 favor the 1,4-dihydroxynonene mercapturic acid (DHN-MA) [349]. The authors compared three different urinary biomarkers of LPO during bromotrichloromethane induced oxidative stress in rats: MDA, the isoprostane 8-iso-PGF2α and DHN-MA as the major HNE urinary metabolite. The rats received a single dose of BrCCl_3_ *per os* and LPO was estimated every day for a 4-day-period. All three parameters significantly increased in response to oxidative stress. DHN-MA showed the highest increase during the 24–48 h period after treatment.

The quantitation of the glutathione conjugate of HNE (HNE-GSH) in the human post-mortem brain was reported by Volkel *et al.*, 2006 applying the specific and very sensitive method of electrospray ionization triple quadrupole mass spectrometry (ESI-MS-MS) [350]. Levels of HNE-GSH conjugates were determined in hippocampus, entorhinal cortex, substantia innominata, frontal and temporal cortex, as well as cerebellum from patients with Alzheimer’s disease and controls matched for age, gender, post-mortem delay and storage time. The levels of HNE-GSH determined ranged between 1 and 500 pmol/g fresh weight in the brain areas examined. The brain specimen from patients with clinically and neuropathologically probable Alzheimer’s disease diagnosed showed increased levels of HNE-GSH in the temporal and frontal cortex. Classification of disease severity revealed highest levels of HNE-GSH in the substantia innominata and the hippocampus, two brain regions known to be preferentially affected in Alzheimer’s disease [4,9,350].

### 7.8. Further Medical Applications of HNE Metabolism

Several studies indicate that the glutathione conjugates of HNE metabolism are mediators of cell signalling and growth. A modification of the metabolic pattern, possibly by pharmacological interventions, could be of therapeutical importance [4]. Addition of HNE or the cell-permeable esters of HNE-GSH or DHN-GSH to cultures of rat aortic smooth muscle cells stimulated protein kinase C, NFκB, and AP-1, and increased cell growth. The mitogenic effects of HNE, but not HNE-GSH or DHN-GSH, were abolished by glutathione depletion. Inhibition of aldose reductase which catalyzes the reduction of HNE-GSH to DHN-GSH prevented protein kinase C, NFκB, and AP-1 stimulation and the increase in cell growth caused by HNE and HNE-GSH, but not DHN-GSH. Furthermore, the growth stimulating effect of DHN-GSH was enhanced in cells treated with antibodies directed against the glutathione conjugate transporters RLIP76 or the multidrug resistance protein-2 [351].

The demonstration that aldose reductase mediates endotoxin-induced inflammation and cardiomyopathy suggests that inhibition of this enzyme may be useful to attenuate maladaptive host responses and to treat acute cardiovascular dysfunction associated with endotoxic shock [335,352]. Srivastava *et al.*, 2006 also demonstrated a contribution of aldose reductase to diabetic hyperproliferation of vascular smooth muscle cells [353]. The authors showed that aldose reductase inhibitors such as tolrestat or sorbinil or antisense aldose reductase mRNA prevented hyperproliferation of cultured rat aortic smooth muscle cells induced by high glucose levels. The results suggest that inhibition of aldose reductase prevents glucose-induced stimulation of smooth muscle cell growth. It was therefore concluded that even though inhibition of aldose reductase increases vascular oxidative stress, this approach may be useful in preventing abnormal smooth muscle cell growth in vessels of diabetic patients.

Tammali *et al.* [354] showed that treatment of the human colon cancer cell line Caco-2 with HNE or HNE-GSH or the aldose reductase-catalyzed product of HNE-GSH, DHN-GSH, resulted in increased cyclooxygenase-2 (COX-2) expression and prostaglandin E2 production. Inhibition of aldose reductase prevented HNE- or HNE-GSH-induced but not DHN-GSH-induced up-regulation of COX-2 and PGE2. The finding that aldose reductase inhibition by siRNAs completely arrested tumor progression in nude mice bearing human colon adenocarcinoma cells suggests that aldose reductase is an obligatory mediator of growth factor-induced up-regulation of COX-2, PGE2, and growth of Caco-2 cells. These findings further suggest that inhibition of aldose reductase may be a novel therapeutic approach in preventing the progression of colon cancer [354].

Another oncological application of modulation of HNE metabolism was suggested in relation to the inhibition of cytosolic class 3 aldehyde dehydrogenase (ALDH3) by antisense oligonucleotides in hepatoma cells [59]. Several changes in aldehyde dehydrogenase isoenzyme expression take place in hepatoma cells. In particular cytosolic ALDH3, not expressed in normal hepatocytes, appears and increases with the degree of deviation. It has been demonstrated that cytosolic ALDH3 is important in determining the resistance of tumor cells to antitumor drugs, such as cyclophosphamide. Moreover, hepatoma-associated ALDH3 seems to be important in metabolizing aldehydes derived from LPO, and in particular the cytostatic aldehyde HNE. It was demonstrated previously that restoring endogenous LPO in hepatoma cells by enriching them with arachidonic acid causes a decrease of mRNA, protein and enzyme activity of ALDH3 and that this decrease reduces cell growth and/or causes cell death, depending on basal class 3 ALDH activity [59]. Applying antisense oligonucleotides the authors were able to demonstrate that ALDH3 mRNA and enzyme activity can be decreased by 90%. Concomitantly cell growth was reduced by about 70% in the JM2 hepatoma cell line with high ALDH3 activity [59].

The carbonyl scavenger *N*-acetylcysteine (NAC) is a pharmaceutical drug and nutritional supplement used primarily as a mucolytic agent and in the management of paracetamol (acetaminophen) overdose. It was used in various investigations to attenuate HNE-mediated organ damage, such as in alcoholic liver damage [355]. In these investigations NAC decreased enzyme release from damaged organs, decreased inflammatory reactivity and prevented significant loss of organ GSH content. NAC exerts also significant protective effects against HNE-induced neuronal death in cerebellar granule neurons. This neuroprotective effect is due, at least in part, to preservation of mitochondrial membrane potential and intracellular GSH levels [356]. An important medical application of modification of HNE metabolism is the development of therapeutic strategies for the prevention of a number of inflammatory diseases [33].

Further studies showed that caloric restriction in mammals and in flies (*Drosophila*) slows the age-related and the oxidative damage-related HNE accumulation [357]. Additionally suppression of apoptosis in aged kidney—where increased HNE and MDA levels were found—by caloric restriction was observed [358]. Additionally, a decrease of oxidative damage in skeletal muscle was demonstrated after caloric restriction of rhesus monkeys [359], and a decline of aldehyde dehydrogenase and glutathione S-transferase activities as pathways of mitochondrial HNE metabolism was prevented in dietary restricted animals [316].

## 8. Conclusions

This review tries to demonstrate that there is a multitude of possible targets of HNE within a cell. Several factors can be envisaged which determine the actual reactants within a cell: The concentration of HNE which can differentially modulate cell death, growth and differentiation [360], the concentration and nucleophilicity of the target, the polarity of the microenvironment, the accessibility within the tertiary structure, and the site of formation as well as the metabolic rate of HNE.

## Abbreviations

Aβ	amyloid beta peptide
ACC	acetyl CoA carboxylase
AcLDL	acetylated low-density lipoprotein
ACO2	mitochondrial aconitase
Acr-dG	adduct of deoxyguanosine and acrolein
AD	Alzheimer disease
ADH	alcohol dehydrogenase
ADP-iron	adenosine diphosphate-iron
AICAR	5-aminoimidazole-4-carboxyamide ribonucleoside
AIDS	acquired immuno-deficiency syndrome
AIE	approximate intracellular concentration (extinction)
AKR	aldo-keto reductase
ALD	alcoholic liver disease
ALDH	aldehyde dehydrogenase
Al-PEs	aldehyde-modified PEs
ALS	amyotrophic lateral sclerosis
AMD	age-related macular degeneration
AMPK	AMP-activated protein kinase
ANT	adenine nucleotide translocase
ApoB	apolipoprotein B-100
AR	aldose reductase
ARE	antioxidant response element
BAG3	Bcl-2- associated athanogene 3
BER	base excision repair
BSA	bovine serum albumin
BSO	l-buthionine-S,R-sulfoximine
CAR	carnosine
CCl_4_	carbon tetrachloride
CERAD	consortium to establish a registry for Alzheimer disease
CH_2_Cl_2_	dichloromethane
CHO	Chinese-hamster ovary
CHOP	C/EBP homologous protein
Col II	collagen type II
COX-2	cyclooxygenase-2
CYP	cytochrome P450
ɛ-dA	1,*N*(6)-etheno-2'-deoxyadenosine
DHN	dihydroxynonene, non-2-ene-1,4-diol
DHN-MA	dihydroxynonene-mercapturic acid
DNA	desoxy-ribonucleic acid
DNA-PK	DNA-dependent protein kinase
DOX	doxorubicin
D3T	3H-1,2-dithiole-3-thione, inducing detoxification enzymes
eEF-2	Eukaryotic Elongation factor 2
EGFP	enhanced green fluorescent protein
EGFR	epidermal growth factor receptor
EHN	2,3-epoxy-4-hydroxy-nonanal
ERK1	extracellular signal-regulated kinase 1
ESI-MS-MS	electrospray ionization triple quadrupole mass spectrometry
FABP	fatty acid-binding protein
FAK	α1β1-integrin-focal adhesion kinase
FID	flame ionization detection
FITC	fluorescein isothiocyanate
GAPDH	glyceraldehyde-3-phosphate dehydrogenase
GC	gas chromatography
GC/MS	gas chromatography/mass spectrometry
GERD	gastroesophageal reflux disease
GRP78	glucose-regulated protein 78
GRX	glutaredoxin
GSH	glutathione
GSH-DHN	glutathione-dihydroxynonene conjugate
GSK3β	glycogen synthase kinase-3-β
GSSG	glutathione in the oxidized form, glutathione disulfide
GST	glutathione-S-transferase
HA-1	Chinese hamster fibroblast control cells
HAEC	human aortic endothelial cell
Hb	hemoglobin
HCC	hepatocellular carcinoma
HCV	hepatitis C virus
HMEC-1	immortalized human microvascular endothelial cells
HHE	4-hydroxy-hexenal
[^3^H]HNE	tritium labeled hydroxynonenal
HNA	hydroxynonenoic acid
HNA-GSH	hydroxynonenoic acid-glutathione conjugate
HNE	4-hydroxy-nonenal
HNE-dG	1,*N*-2-propane adduct of guanine and HNE
HNE-H2A	HNE-modified histone-H2A
H2O2	hydrogen peroxide
HPLC	high performance liquid chromatography
HPNE	4-hydroperoxy-2-nonenal
HO-1	heme oxygenase-1
HSA	human serum albumin
HU	HU hydroxyurea
HUVEC	human umbilical vein endothelial cell
ICAM-1	intercellular adhesion molecule-1
IsoLGs	isolevuglandins
iTRAQ	isobaric Tags for Relative and Absolute Quantitation
LA	linolenic acid
LAD	late Alzheimer disease
LADH	lipoamide dehydrogenase
LCA	retinal dystrophy Leber congenital amaurosis
LC-MS/MS	liquid chromatography-tandem mass spectrometry
LDH	lactate dehydrogenase
LDL	low density lipoprotein
LKB1	liver kinase B1
LOX1	lectin-like oxidized low-density lipoprotein receptor-1
LPO	lipid peroxidation
MAL-6	2,2,6,6-tetramethyl-4-maleimidopiperidin-1-oxyl
MALDI-TOF	matrix-assisted laser desorption/ionization-time of flight
MAPK	mitogen-activated protein kinase
MBP-1	c-myc binding protein-1
MCI	mild cognitive impairment
MDA	malondialdehyde
MDM2	mouse double minute 2
MH1C1	rat hepatoma cells
MMP-13	matrix metalloproteinase 13
MRP-1	multidrug resistance protein-1
NAD+	nicotinamid dinucleotide, oxidized form
NADH	nicotinamid dinucleotide, reduced form
NADP	nicotinamid dinucleotide phosphate
NAFLD	non-alcoholic fatty liver disease
NEP	neprilysin
NER	nucleotide excision repair
Nrf2	NF-E2-related factor 2
OA	osteoarthritic
OC5 and OC14	Chinese hamster fibroblast H_2_O_2_-resistant cell lines
ONE	4-oxo-nonenal
oxLDL	oxidized low-density lipoprotein
ox-PAPC	oxidized PAPC
PAF	1-*O*-alkyl-2-acetyl-sn-glycero-3-phospho-choline
PAPC	1-palmitoyl-2-arachidonoyl-*sn*-glycero-3-phospho-choline
PCAD	preclinical Alzheimer disease
PDGF	platelet-derived growth factor
PDI	protein disulfide isomerase
PE	phosphatidyl-ethanolamine
PGE	prostaglandin E
Pin1	peptidyl-prolyl *cis/trans*-isomerase A1
PMF	peptide mass fingerprinting
POS	photoreceptor outer segment
PG(E2)	prostaglandin E2
PtdIns(3,4,5)P-3	phosphatidylinositol-3,4,5-trisphosphate
PTEN	phosphatase and tensin homolog deleted on chromosome 10
RAS	rennin/angiotensin system
RBC	erythrocytes
RDH	retinol dehydrogenase
RLIP76	Ral-binding GTPase activating protein
ROC	receiver operator curves
ROOH	lipid hydroperoxide
ROS	reactive oxygen species
RPE	retinal pigment epithelial
SAM	sterile-alpha motif
SCFAs	short chain fatty acids
SERCA1a	serco/endoplasmic reticulum Ca^2+^-ATPase
SIRT3	sirtuin 3
SLE	systemic lupus erythematosus
SMC	smooth muscle cell
sXBP1	spliced X-box-binding protein-1
α-Syn	α-synuclein
TBA-RS	thiobarbituric acid-reactive substances
TBI	traumatic brain injury
TLC	thin layer chromatography
TLR	toll-like receptor
TRPV1	transient receptor potential vanilloid 1
TRX	thioredoxin
TrxR	thioredoxin reductase
UCP	uncoupling protein
UPR	unfolded protein response
VSL#3	a medical food delivering high amount of beneficial live bacteria
YAP1	Yeast Activator Protein 1
ZAK kinase	sterile alpha motif and leucine zipper containing kinase (AZK)
